# H/C atomic ratio as a smart linkage between pyrolytic temperatures, aromatic clusters and sorption properties of biochars derived from diverse precursory materials

**DOI:** 10.1038/srep22644

**Published:** 2016-03-04

**Authors:** Xin Xiao, Zaiming Chen, Baoliang Chen

**Affiliations:** 1 Department of Environmental Science, Zhejiang University, Hangzhou 310058, China; 2 Zhejiang Provincial Key Laboratory of Organic Pollution Process and Control, Hangzhou 310058, China; 3 Department of Environmental Engineering, Ningbo University, Ningbo 315211, China

## Abstract

Biochar is increasingly gaining attention due to multifunctional roles in soil amelioration, pollution mitigation and carbon sequestration. It is a significant challenge to compare the reported results from world-wide labs regarding the structure and sorption of biochars derived from various precursors under different pyrolytic conditions due to a lack of a simple linkage. By combining the published works on various biochars, we established a quantitative relationship between H/C atomic ratio and pyrolytic temperature (*T*), aromatic structure, and sorption properties for naphthalene and phenanthrene. A reverse sigmoid shape between *T* and the H/C ratio was observed, which was independent of the precursors of biochars, including the ash contents. Linear correlations of Freundlich parameters (*N*, log *K*_f_) and sorption amount (log *Q*_e_, log *Q*_A_) with H/C ratios were found. A rectangle-like model was proposed to predict the aromatic cluster sizes of biochars from their H/C ratios, and then a good structure-sorption relationship was derived. These quantitative relationships indicate that the H/C atomic ratio is a universal linkage to predict pyrolytic temperatures, aromatic cluster sizes, and sorption characteristics. This study would guide the global study of biochars toward being comparable, and then the development of the structure-sorption relationships will benefit the structural design and environmental application of biochars.

Biochar, a carbon-rich material that is produced from biomass pyrolysis under little or no available air at a relative low temperature (<700 °C)^1^,^2^,^3^,^4^,^5^,^6^, is attracting increasing attention as a carbon-negative product^1^ and as an effective adsorbent for environmental and agricultural applications^3^,^7^,^8^. For example, age-old and carbon-rich *Terra Preta* soil in the Amazon basin was used to sequester carbon for millennia through anthropogenic amendment of charred organic matter^1^,^4^,^9^. Afterward, a series of studies on the applications and effects of biochar on soil ecosystems was conducted in the field of agriculture and environment. Biochar provides many benefits for agricultural production, such as improving soil CEC^5^, alleviating soil aluminum phytotoxicity^10^,^11^, contributing silicon fertilizer^12^, retaining nutrients^13^, and promoting electron transfer^14^ in soil. Biochar was found to have controllable structures for sorption^3^ and would play a vital role in the fate of pollutants via altering their mobility and bioavailability^15^,^16^,^17^. To date, numerous researchers have paid more attention to the sorption behavior of various pollutants onto diverse biochars^3^,^18^,^19^. However, it is a large challenge to compare the reported results from world-wide labs regarding the structure and the sorption of biochars derived from various precursors under different pyrolytic conditions and pretreatments because of the absence of a unified sample or a well-accepted linkage between pyrolytic temperatures, structures, and sorption properties of various biochars. Global interests on biochars urgently require solving this challenge.

In fact, the vital factor that influenced sorption capacity was the biochar structure, which was an integrated reflection of all of the different preparation processes and treatments, and the development of a structure-sorption relationship can offer a potential way to solve the problem of comparison. Due to its heterogeneous nature, biochar structure was distinguished between non-carbonized matter and carbonized matter, which acted as a partition phase and adsorbent, respectively^3^. An isotherm-separation method was used to quantitatively calculate the contribution of partition and adsorption^3^. These analysis further declared a transitional sorption mechanism from partition-dominant at low pyrolysis temperature to adsorption-dominant at high pyrolysis temperature for biochar^3^, which was extensively confirmed by subsequent studies. Furthermore, the structures of biochar also impact sorption kinetics and sorption thermodynamics^7^,^20^,^21^. With increasing pyrolytic temperatures, a reduction of polar functional groups and a formation of aromatic cores in the biochars were observed^3^. A similar structural transformation of biochars with heating temperature was demonstrated by several studies^7^,^12^,^20^,^22^. Keiluweit *et al*.^22^ categorized four distinct char phases and physical states transited with increasing pyrolytic temperature, including transition chars, amorphous chars, composite chars, and turbostratic chars. Brewer *et al*.^23^ proposed some estimated molecular structures of biochar via fast pyrolysis, slow pyrolysis and gasification systems by quantitative ^13^C nuclear magnetic resonance spectroscopy (^13^C-NMR). These results expanded our knowledge of biochar structures. Nevertheless, the specific structure of various biochars still remains unknown. Actually, the enormous variety of types of precursor matter (grass^24^, wood^25^, rice straw^12^,^26^, rice bran^27^, dairy manure^8^,^28^, and chicken manure^28^), different preparation methods (slow pyrolysis^3^, fast pyrolysis^23^, gasification^23^, and others such as hydrothermal^29^,^30^ and microwave pyrolysis^31^,^32^,^33^), and different heating temperatures (100–700 °C) made the structures of biochar quite various. Therefore, the structure-sorption relationship based on various biochars from different labs has not been developed.

As reported, pyrolytic temperature plays a more important role in shaping the structure and the sorption of biochar^3^,^22^,^34^. When the charring temperature was lower than 500 °C, the H-bonding network in lignocellulose was eliminated, and hydroxyl groups were oxidized to carboxyl^34^. Methylene groups were heavily dehydrogenated when the charring temperature was higher than 500 °C^3^,^22^,^34^. Recently, thermodynamic processes in the pyrolysis conversion of biomass and manures to biochars indicated overlapping pyrolysis temperature ranges of cellulose, hemicellulose and lignin in the precursor feeds, which can be separately quantified by peak fitting from thermogravimetric analysis^28^. Despite the variation of precursors and preparation methods, the cellulose, hemicellulose and lignin components in feedstocks experienced similar chemical bond cracking and formation processes with the pure components^26^, which may make the structure of the organic fractions of various biochars comparable to some extent. Theoretically, there may be a hidden rule connecting the pyrolytic temperature and the sorption behaviors of various biochars. Thus, a linkage index is urgently needed to represent the physicochemical properties of biochars and then to build a quantitative relationship with their sorption behaviors.

H/C atomic ratio seems to be a perfect mediate index for reflecting the aromaticity of biochars and for predicting the sorption of hydrophobic organic contaminants (HOCs) onto biochars. A linear relationship between H/C and the Freundlich fitting parameters was reported^3^. Han *et al*.^35^ declared a positive relationship between the aromaticity of biochars and their sorption of phenanthrene. In addition, HOC sorption was mainly contributed from aromatic moieties in natural organic matter^36^,^37^,^38^,^39^, but no quantitative relationship between H/C and sorption capacity of various biochars and the associated mechanisms was systematically revealed. The current challenge of the updated study is that we know the effect between the charring temperature (T), the H/C atomic ratio, aromaticity and sorption capacity, but hardly do we know the quantitative relationship and mechanism between them, as it was shown in the main scheme of the current study in Fig. 1.

To achieve these quantitative relationship and make them more convincing and representative, we collected hundreds of published data from our previous works and others regarding different pyrolytic temperatures, various feedstocks and structural characteristics of biochars, and sorption properties of naphthalene and phenanthrene as model HOCs. According to Johannes Lehmann and Stephen Joseph’s suggestions, biochar as a commercial product is defined as “the product of heating biomass in the absence of or with limited air to above 250 °C, which is intended for use as a soil application or broader for environmental management”^40^. But in the fundamental study on biochar’s structure and properties, biochar is considered as a pyrogenic residue under a relative low and continuous temperature (<700 °C)^1^,^2^,^3^,^4^,^5^,^6^,^7^,^12^,^20^,^22^,^23^. Therefore, in the current study the biochars prepared under wide temperature range were collected to recognize the linking rule of structures and properties of various biochars. The hydrochar (the solid product of hydrothermal carbonization or liquefaction), charcoal (produced by thermochemical conversion from biomass for energy generation), activated carbon (the pyrogenic carbonaceous material has undergone activation), soot (the component resulting from gas condensation process) are generally not considered to be biochars here. The high temperature derived biochars (HTB) is referred to pyrolysis residue of feedstock at heating temperature >400 °C, which mainly experience the aromatization processes^5^,^12^,^28^. A quantitative relationship between the charring temperature (T) and the H/C atomic ratio, between H/C and aromatic cluster size, and between sorption properties and H/C was hopefully achieved.

## Results and Discussion

### Quantitative relationship between pyrolysis temperature and H/C atomic ratio. 

**Figure 2:**
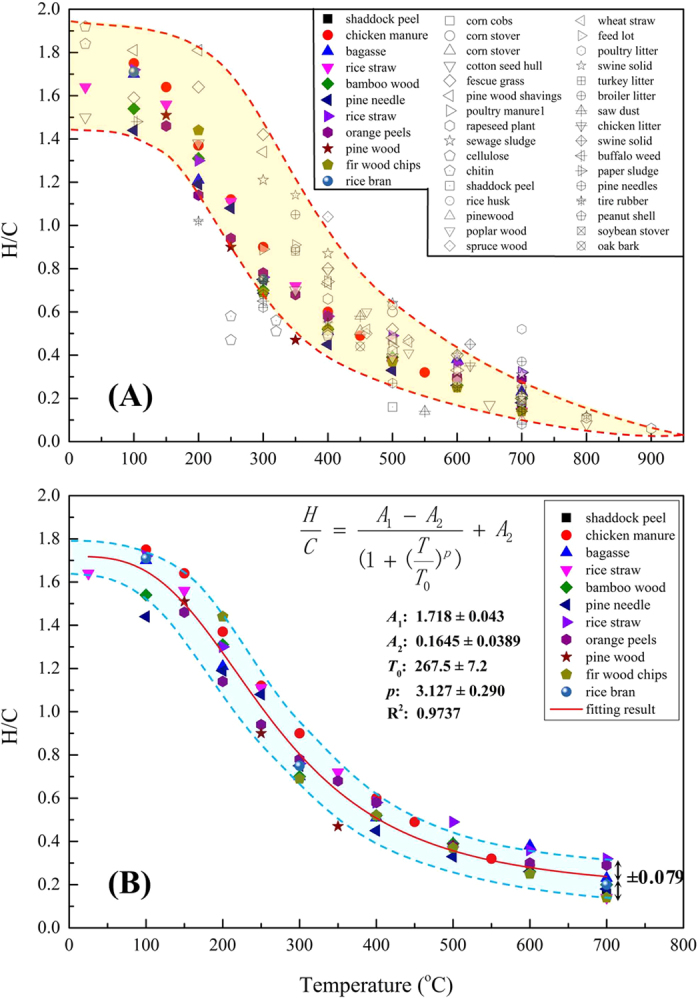
The relationship between the pyrolysis temperature (*T*) and H/C atomic ratio of various biochars derived from different precursors. (**A**): all of the cited data, and (**B**): the data from our lab. The points with solid symbols were cited from our lab, and the points with hollow symbols were cited from other groups. The detailed data could be found in Table S-1.

The relationship between the heating temperature (*T*) and the H/C atomic ratio of various biochars from both our lab and others was demonstrated in Fig. 2A. Clearly, the H/C atomic ratio generally decreased with heating temperature, which was consistent with the reported increasing aromaticity because H/C was an index of aromaticity^3^,^22^. Although the precursors (42 feedstocks) varied greatly, including biomass, manures, and even sludge, there were still some inherent laws when they experienced the same oxygen-limited pyrolysis process. The shadow area in Fig. 2A indicated that the H/C range at a certain pyrolysis temperature became narrow with increasing pyrolysis temperature, suggesting that the similar molecular structures may be formed at relative high pyrolysis temperature.

Interestingly, a relative regular curve between *T* and H/C was obtained in Fig. 2B after the preparation method was unified to the heating rate of 5 °C/min and holding time of 6 h (Table S-1 in Appendix A. Supplementary data). The shadow area in Fig. 2B showed the varying trend of H/C with charring temperature, which looked like a reverse “S” type curve. Despite the tremendous difference in the precursor structures and the mineral constituents (pine needle^3^ had less than 1% ash, whereas that of rice straw was higher than 16.7%), the consistency of *T* ~ H/C collectively shown indicated the relatively coherent transformation of biochars with charring temperature. Therefore, it is possible to unify the structure model of biochars derived from different precursors under specific heating temperatures. The consistency actually came from the similar pyrolysis processes that the organic matter in precursors experienced, including dewatering at 0–150 °C, hemicellulose/cellulose/lignin cracking at 200–400 °C, and aromatization at 400–700 °C^12^,^28^,^41^. The points from other research groups that deviated from the shadow area of Fig. 2B were prepared using different methods. After summarizing their preparation procedures, we believed that the holding time at a charring temperature made a substantial difference. The corresponding heating rate and holding time was given in Table S-1. The longer the heating temperature was held, the more adequate the pyrolysis reactions that the precursors went though, further resulting in a lower H/C value^3^,^22^,^42^,^43^. For instance, according to Fig. 2A, fescue grass and pine wood shavings^22^ (the hollow symbols, holding time is 1 h) showed distinctly higher H/C values than those of precursors with a holding time of 6 h (the solid symbols) when the heating temperature was 200~400 °C. Additionally, the biochars derived from cellulose and chitin^44^ had much lower H/C values with a holding time of 8 h. The regular reverse sigmoid curve of H/C value varying with *T* suggests that pyrolytic temperature plays a more important role in shaping biochars compared with the preparation method and the type of precursors. This finding will guide the design of biochar materials with tunable structures from different precursors and optimize their environmental applications as soil sorbent and carbon sequestration strategy.

Moreover, a logistic functional formula was found to have an unexpected good fitting result on the *T*~H/C curve. The corresponding formula, parameters and fitting results were given in Fig. 2B. The function formula was:


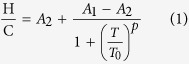


where *T* represented the heating temperature (°C);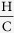 represented the H/C atomic ratio (dimensionless). The parameters *A*_*1*_, *A*_*2*_, and *p* were dimensionless constants; *T*_0_ was a constant (°C). The fitted result was written as:


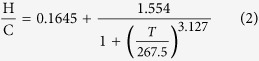


This quantitative relationship between *T*~H/C is the first reported, but the physical significance of those constants could also be analyzed. First, it is a decreasing function, implying that the H/C value decreases with heating temperature. Second, when *T/T*_0_ ≤ 0.1, 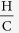 is approximately unchanged and approaches *A*_*1*_ = 1.72, indicating that the original precursor commonly has a H/C value of 1.72. This number is very close to the H/C value of 1.67 in cellulose ((C_6_H_10_O_5_)_n_), which is the basic component in biomass. Third, when *T/T*_0_ ≥ 10, 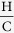 approaches *A*_*2*_, meaning that the H/C is close to 0.165 at high charring temperature. The lack of data for biochar derived at higher pyrolysis temperatures may be attributed to the reason that led to the unreasonable fitting result of *A*_2_ being 0.165: that H/C theoretically trends toward zero as the heating temperature increases. For example, the H/C ratio in graphite formed at a temperature higher than 2000 °C should be theoretically close to 0. Fourth, *T*_0_ is the temperature of 267.5 °C, where the rate of decrease of the H/C ratio was the highest, meaning that *T*_0_ refers to the charring temperature where the precursor was cracked most intensely. Finally, the parameter *p* represents the difficulty level for aromatization of the precursors. In other words, it referred to the heating temperature level that was needed to achieve the same degree of aromatization. A longer holding period usually brought about higher *p* values, which means that to achieve the same H/C value, the heating temperature was lower for a longer holding period. Conversely, a much higher temperature would be required to obtain the same H/C value for the fast pyrolysis. Although the data used to establish this *T* ~ H/C equation generally came from the same preparation procedures, further modifications may be expected to be made for the data from different preparation methods. The charring temperature could be inversely calculated by testing the H/C atomic ratio, which was useful for predicting the properties of naturally-formed black carbon (a kind of pyrogenic carbonaceous material similar to biochar, but dispersed in the environment from wildfires and fossil fuel combustion^40^). For example, the H/C atomic ratio of natural black carbon was measured in the range of 0.23~0.76^45^. Therefore, the corresponding heating temperature of that black carbon was calculated in the range of 312~700 °C, which was consistent with the suggested temperature (280~500 °C) that black carbon was formed at.^46^ Although the inaccuracy can be minimized by further modification, as far as we know, this is the first report that provides a quantitative relationship between the charring temperature and H/C values of various biochars derived from different precursors and preparation methods.

### Quantitative correlation of H/C atomic ratio with Freundlich parameters

**Figure 3:**
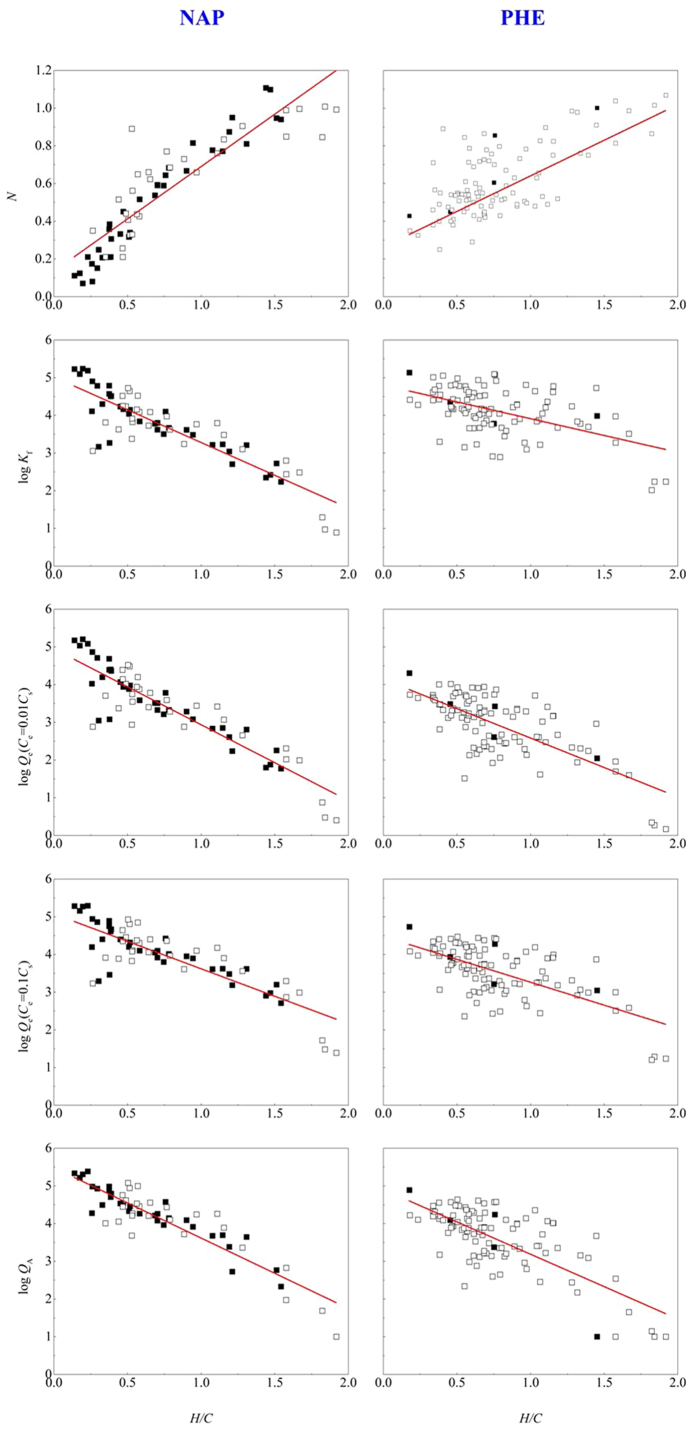
The correlation of H/C atomic ratio with Freundlich parameters (*N,* log *K*_f_), log *Q*_e_ (*C*_e_ = 0.01*C*_s_), log *Q*_e_ (*C*_e_ = 0.1*C*_s_) and log *Q*_A_ of NAP and PHE sorption to biochars. The solid symbols represent the data sets from our research group, while the hollow symbols from the others. The detailed data can be found in Table S-2 for NAP and Table S-3 for PHE. Fitted results are given in Table S-4. Noting that 35 out of 65 data sets on NAP sorption, and 5 out of 98 data sets on PHE sorption were from our research group. *Q*_e_ is the equilibrium adsorbed quantity, mg kg^−1^; *C*_e_ is the equilibrium concentration, mg L^−1^; *Q*_A_ is the calculated maximum adsorption capacity, mg kg^−1^; *C*_s_ represents the sorbate solubility in water, mg L^−1^.

The relationship between the aromatic index of biochar and the corresponding sorption parameters would benefit the prediction of sorption behavior of aromatic pollutants onto biochars. Freundlich fitting on the sorption isotherm has relatively good results over the full-range of heating temperatures^3^. A linear relationship between H/C atomic ratio and the Freundlich parameters (*N* index and log*K*_f_) has been noticed for naphthalene, nitrobenzene and m-dinitrobenzene sorption to biochars derived from pine needles and rice straw^3^,^47^. However, this was limited to biochars derived from just one precursor. In the current study, we collected the reported sorption data of NAP and PHE onto biochars derived from various precursors (Fig. 3).

In Tables S-2 and S-3, the precursor feedstocks, preparation methods, elemental contents, and ash contents of biochars and the corresponding Freundlich parameters for the sorption of NAP and PHE were provided. All of the Freundlich constants of log*K*_f_ were ash-corrected to focus on the effect of the organic matter. Figure 3 showed the correlation of H/C ratio with the sorption parameters, and the fitted results of those correlations were listed in Fig. S-4. For both NAP and PHE, it could be found that the parameter *N* generally increased as log *K*_f_ decreased with increasing H/C atomic ratio, indicating that the higher the aromaticity of the biochar, the lower the *N* and the higher the log *K*_f_ would be. A correlation test was conducted on the relationship between H/C atomic ratio and Freundlich parameters by Pearson’s test using the SPSS software. The fitting results indicated that the H/C atomic ratio was linearly correlated with *N* index and log *K*_f_ with high significance, and the Pearson correlation coefficients (R) were 0.8990 and −0.8735 for NAP (**p < 0.01, *n* = 65, two-sided test) with a statistical significance at the level of **p < 0.01 (|R| > 0.313, *n* = 65), and 0.6915 and −0.5300 for PHE (**p < 0.01, *n* = 98, two-sided test) with a statistical significance at the level of **p < 0.01(|R| > 0.256, *n* = 98). The significantly quantitative relationships between the H/C atomic ratio and the Freundlich parameters were written below:


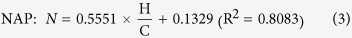



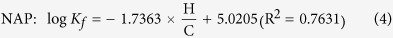



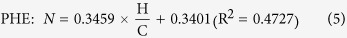



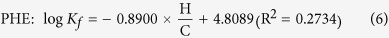


Because the data presented in Fig. 3 contained both biomass-derived and manure-derived biochars, and even chemically treated biochars, these linear fitting results suggested that the sources of biochars and the preparation procedures had little effect on the crucial correlation of Freundlich parameters (*N* and log *K*_f_) with the H/C atomic ratio of biochar. Noting that 35 out of 65 data sets on NAP sorption, and 5 out of 98 data sets on PHE sorption were from our research group. Thus, these linear relationships between Freundlich parameters with the H/C atomic ratio are universal. Comparing the fitting results of NAP and PHE, it is interesting to find that the relationships of H/C *~ N* and H/C ~ log*K*_f_ exhibit a much better fitting result for NAP than for PHE. This was not only because of more data groups for PHE sorption, more diverse precursor feedstocks and more treatment methods for PHE but also due to the larger molecular size of PHE than of NAP, which made PHE easily induce the size effect. Another thing to be noticed is that the *T*~H/C relationship mentioned above can be affected by the holding time of the pyrolysis process, whereas the relationship between H/C and the Freundlich parameters seemed to not be affected by the holding time or by the precursors. It is an important sign that the preparation procedures (with the different heating temperatures and holding times) shape the structure of biochars, which is reflected by the values of H/C and the H/C ratio being linearly linked with Freundlich parameters (reflected sorptive characteristics), which suggests that a potential structure-adsorption relationship for various biochars derived from different feedstocks may exist.

In fact, the *N* index and log *K*_f_ were linearly dependent on H/C ratios, and the logarithmic sorbed amount (log *Q*_e_) was calculated linearly from *N*, log *K*_f_ and the logarithmic equilibrium concentration (log *C*_e_) according to the linear form of the Freundlich equation. Therefore, the linearity between H/C and log *Q*_e_ inevitably exists in theory when *C*_e_ is specified. As shown in Fig. 3 and Table S-4, a linear relationship was also found between the H/C ratio and log *Q*_e_ at *C*_e_ = 0.01*C*_s_ and 0.1*C*_s_, meaning that H/C may be used as a smart and simple parameter for predicting the total sorption capacity of various biochars.

### An aromatic cluster prediction model of biochars based on the H/C atomic ratio

For the high temperature derived biochars (HTB) (>400 °C), the precursors mainly experienced the aromatization processes and generated tiny aromatic cluster graphene-like structures^5^,^12^,^28^. The nuclear magnetic resonance (^13^C-NMR) had confirmed that carbon in high temperature-derived biochar was mostly aromatic^48^. Further specific quantitative NMR techniques by Brewer *et al*.^23^ estimated the size of the aromatic clusters in biochars, which was affected by preparation methods. The solvent-extractable PAHs (e.g., benzo [g,h,i] perylene in HTB) were detected, which contained six rings (the highest detectable number)^49^, confirming the existence of a fused ring structure in HTB. Thus, there may be more rings in aromatic hydrocarbons acting as the basic structure in HTB.

**Figure 4:**
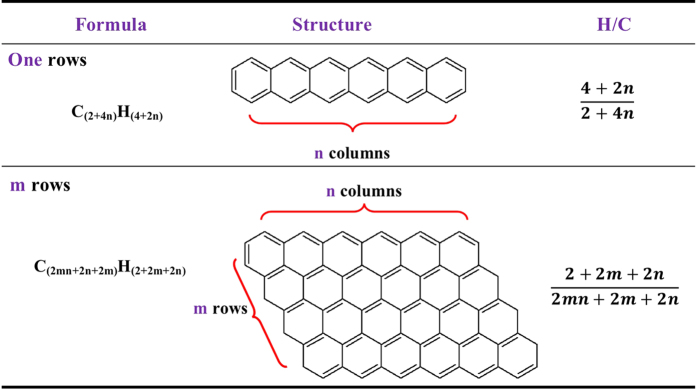
Structural illustration of the aromatic cluster of biochars based on the proposed model of the rectangle-like aromatic ring.

In consideration of the H/C ratio being an aromatic index, we initially used it to estimate the size of aromatic clusters which would act as the structure model for HTB. The reasons why the H/C atomic ratio was chosen are as follows: (1) The H/C atomic ratio is a quantity obtained by taking biochar as a whole and is easily acquired from elemental analysis. The structure model calculated by the H/C atomic ratio could be valuable and representative of HTB. (2) The organic matter in HTB contains mainly carbon and hydrogen, and the H/C atomic ratio was relatively accurate compared with other atoms ratios. For example, the oxygen content was calculated by ash correction, which could contribute substantial error, and other atoms had negligible atomic content. (3) The H/C atomic ratio is often connected to the O/C ratio in van Krevelen plots^50^. By connecting H/C and O/C, the different characteristic organic matter precursors were distinguished, and different variations of H/C ~ O/C relationships reflected different reactions, among which oxidation and reduction would change the O/C value but not the H/C value^50^. Thus, the H/C ratio becomes a relatively unchanged index during the aging process of biochar. Additionally, O/C is often taken as the index to evaluate the degree of oxidation and stability of biochar^51^. The H/C atomic ratio seems to be more stable than other atomic ratios, even during the oxidizing process because the oxidation of aromatic hydrocarbon (alkane) into phenol (alcohol) changed the O/C atomic ratio but not the *H/C* atomic ratio, for instance. Based on the analysis above, we assumed that (1) the effect of other atoms (O, N, S…) on the structure of HTB could be ignored; and (2) all of the carbon and hydrogen atoms existed in the aromatic layer fractions. The distribution of fused aromatic rings was supposed to be a rectangle-like pattern. The corresponding structure, formula, and H/C atomic ratio of this rectangle-like pattern were shown in Fig. 4, where *m* represented the row number, and *n* represented the column number.

**Figure 5:**
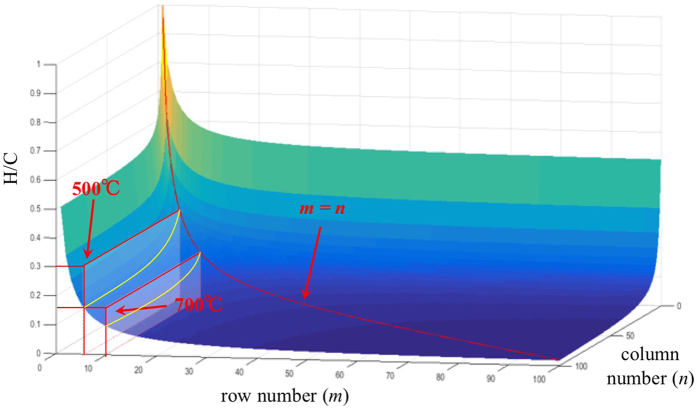
H/C values as a function of *m* and *n* for the aromatic cluster of biochars based on the rectangle-like polycyclic aromatic model. The red curve shows the H/C variation when *m* = *n*.

In the structure of m*n polycyclic aromatic rings, hydrogen atoms are all linked to the marginal carbon, and the theoretical formula was C_(2mn+2n+2m)_H_(2+2m+2n)_. As a result, the H/C value depends on the values of *m* and *n*, which means that the H/C value relates to the size of the aromatic clusters (shown in Fig. 5). When *m* is equal to 1, the H/C atomic ratio was 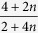, then the limit of the H/C atomic ratio is 
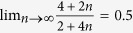
, which is greater than the average value 0.20 of 700 °C derived biochar from rice bran in Table S-1 27. Thus, we set *m* = *n*, and the H/C atomic ratio became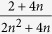. Table 1 shows the corresponding H/C values as a function of *n*, and the red curve in Fig. 5 shows that the H/C value has a one-to-one correspondence to *n*, implying that the H/C value could be used to predict the aromatic cluster sizes of HTBs.

Taking advantage of the H/C value, the molecular structure and aromatic cluster of biochars can be predicted according to Table 1. For instance, Brewer *et al*.^23^ studied the structure of Switchgrass S. P., whose H/C atomic ratio was calculated to be 0.396 according to the published elemental analysis. Hence the corresponding structure of the aromatic cluster was distributed in an approximately 3*3 rectangle, which was well matched with the estimated 8-ring structure from NMR analysis^22^. In addition, the biochar derived from pine needles produced at 500 °C (P500) has a H/C atomic ratio of 0.329^3^, so the aromatic cluster of P500 is a 5*5 rectangle distributed by checking Table 1 (shown in Fig. 5). Analogously, the aromatic cluster of P700 is distributed in a 10*10 rectangle (H/C = 0.176)^3^. Considering that the diameter of a benzene ring in graphene is 0.246 nm, the calculated aromatic cluster size of P500 and P700 should be 1.25 nm*1.25 nm and 2.5 nm*2.5 nm, respectively. The molecular structure calculated by the proposed model using the H/C ratio as an index did not mean that there was a nano-graphene structure in biochar but represented only the integrated skeletal structure by taking biochar as a whole. Simultaneously, we cannot deny the possible existence of such structures in biochar. Some previous studies confirmed the existence of this type of aromatic cluster structure by intuitive skeletal evidence. Atomic-scale transmission electron microscopy (TEM) directly observed the nano-sized crystalline graphene from biochar derived from rice husks (850 °C), which consists mainly of 200–300 carbon atoms having the character of graphene nanosheets^52^, indicating that the benzene ring number of the graphene nanosheet was 100–150, which is approximately in accordance with the 10*10 = 100 rings for 700 °C biochars. These observations provided a direct verification for the predictive model of aromatic clusters of biochars using the H/C ratio as an index. Therefore, it is reasonable and feasible to use the H/C ratio to predict the aromatic skeleton structure of biochars, and moreover this type of calculation yields a new and facile way to develop aromatic clusters in biochars.

### Adsorption capacity of aromatic pollutants linking to the H/C atomic ratio of biochars

The relationship between H/C and log *Q*_e_ has been illustrated, where log *Q*_e_ represented the total sorbed quantity calculated at one specific equilibrium sorbate concentration (*C*_e_) in water. Both partition and adsorption contributed to the total sorption of biochars^3^. Therefore, the maximum adsorption capacity of biochars was required to develop the structure-adsorption relationship to predict the potential adsorption. *Q*_A_ was the right maximum adsorption capacity stemming from the linear fitting of the high concentration isotherm data^3^. Figure 3 offered the relationships between the H/C ratio and *Q*_A_ (fitted results shown in Table S-4). The linear curves between log *Q*_A_ and H/C were achieved (Figure 3). The corresponding fitting results were also given as follows:


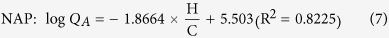



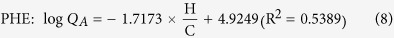


According to Fig. 3, log *Q*_A_ decreased linearly with increasing H/C atomic ratio. The similar aromaticity of NAP and PHE made the linear slope of NAP and PHE almost the same, but the slope of NAP was slightly higher than that of PHE, which may owe to the higher accessibility of NAP than PHE due to the size effect. In addition to the slope that reflected the affinity between aromatic pollutants and aromatic surfaces, the intercept of the fitted linear curve actually represented the highest ideal adsorption capacity of an aromatic surface to a certain pollutant because the intercept was obtained when H/C equaled 0.

**Figure 6:**
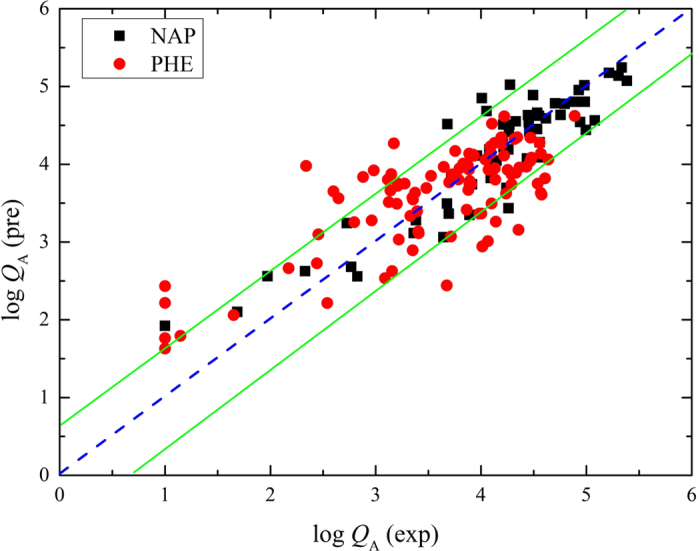
Relationships of log *Q*_A_ (exp) and log *Q*_A_ (pre) of organic pollutants onto biochars. Log *Q*_A_ (exp) represented the experimental logarithmic maximum adsorption capacity generated from the high-concentration linear fitting, which was shown in Tables S-2 and S-3. Log *Q*_A_ (pre) represented the logarithmic maximum adsorption capacity predicted from the fitted linear relationship between H/C and log *Q*_A_. The units of *Q*_A_ are mg kg^−1^.

Figure 6 showed that the experimental log *Q*_A_ values were excellently consistent with the predicted log *Q*_A_ for both NAP and PHE: log *Q*_A_ (pre) = log *Q*_A_ (exp) ± 1.07 (the confidence interval is 95%). The predicted log *Q*_A_ was calculated from the measured H/C value of biochars via equations (7) and (8). The experimental log *Q*_A_ was calculated from the experimental fitted Freundlich parameters. The non-biochar carbon materials were also summarized in Table S-5, including the natural organic materials (such as coal^53^, humic acid^39^,^54^,^55^,^56^, lignite^57^, and sediment^58^) and novel carbon materials (such as carbon nanotubes^59^ and graphene^60^). A good match between the experimental log *Q*_A_ and the predicted log *Q*_A_ demonstrated a universal feasibility of the relationship of H/C ~ log *Q*_A_ (Figure S-2).

**Figure 7:**
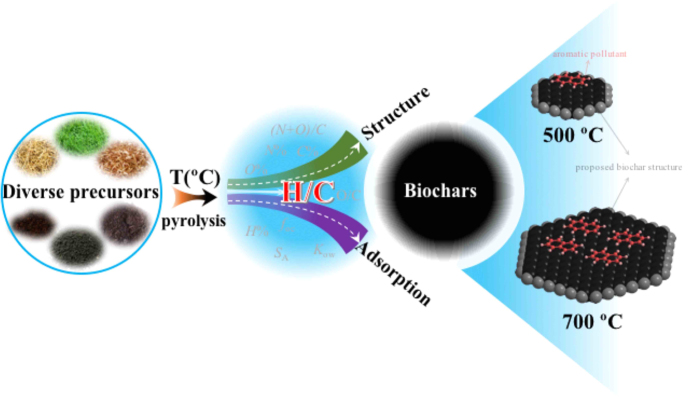
The relationships among pyrolytic temperature, structural characteristics and adsorption properties of biochars derived from diverse precursors via H/C ratio as a universal linkage. This figure describes the core thought of this study, in which four aspects of biochars, such as pyrolysis temperature (T), H/C atomic ratio, adsorption behavior, and molecular structure, were discussed. Of all the four aspects, the H/C ratio acts as a smart linkage to the others, and their quantitative relationships are carefully illustrated in the text. The right molecular model is simply to describe the adsorption behavior of aromatic pollutant onto 500 °C and 700 °C derived biochars, and the adsorption is higher in biochars obtained at 700 °C.

Theoretically, the structure-adsorption relationship can be developed because H/C in equations (7) and (8) was an index of the aromatic cluster structure of biochar, and it was a one-to-one relationship when *m* = *n* according to the rectangle-like polycyclic aromatic ring model. Therefore, the aromatic structure and the NAP (PHE) adsorption capacity of biochar were one-to-one linked. Moreover, adsorption ability was linked with the aromatic cluster sizes of biochars. For instance, biochars derived from pine needles prepared at 500 °C and 700 °C have H/C values of 0.329 and 0.176, respectively, corresponding to 5*5 and 10*10 aromatic cluster sizes as mentioned in the rectangle-likes models. The calculated aromatic cluster sizes implied larger triple aromatic cluster areas of biochars derived at 700 °C than those of biochars derived at 500 °C. According to Table S-2, for precursors such as pine needles^3^, pine wood^20^, maize stalk^61^, bagasse, and bamboo, *Q*_A_ values of various biochars at 700 °C were 4.296, 1.238, 3.504, 2.885, and 3.018 times higher than those of biochars at 500 °C, respectively, which kept a perfect match with the estimated aromatic cluster ratio of biochars (Fig. 7). This type of agreement had a good match with the estimated aromatic cluster and its corresponding adsorption capacity, and the structure-adsorption relationship was actually an inherent reflection of the quantitative mathematical relationship between the H/C ratio and adsorption capacity (Fig. 7). As far as we know, this is the first report to explain the structure-adsorption relationship of biochars in the view of the estimated aromatic cluster sizes with H/C as a mediate parameter at a molecular level, which will lead to the knowledge of sorption mechanisms at a molecular level. The adsorption capacity is only one function of biochar. More studies are wanted to illustrate benefits, risks and challenges of biochar applications^1^,^5^,^6^ and then their linking to the molecular structure of biochars.

## Conclusion

The various precursors, preparation methods, and experimental conditions hinder the comparison of the sorption results from different labs world-wide. The connections of the charring temperature (*T*) ~ H/C, H/C ~ Freundlich parameters (*N*, log *K*_f_), H/C ~ sorption capacity, H/C ~ aromatic clusters sizes, and H/C ~ adsorption capacity were proposed in this study, which strongly suggest that the H/C ratio is a smart linkage between the preparation temperatures, aromatic clusters and sorption properties of biochars. In this study, we are not supposed to say that other factors of biochars (such as pore size, particle size and surface area) have no effect on the sorption. Actually, the pyrolysis temperature matters the most, and H/C ratio value is a reflection of the basic and elemental structure of biochar, which further constitute the different pore size, surface area of biochars. Moreover, if a kind of carbon material sorption capacity is much lower than the estimated value in this study, the reason may be that the ash-correction have not been processed, or that the sorption ability of this material have not been fully released due to some reasons (such as self-agglomeration), or that H/C ratio value was not so precise. These findings not only provide a new way to estimate the aromatic cluster structures of biochars but also offer a novel way to predict the sorption capacity of biochars for aromatic organic pollutants. It is a first very important step to compare and predict the adsorption capacity of different biochar products in a rapid way since H/C ratio value is comparably easy to measure, and will unify the sorption studies of biochars throughout the world, making them comparable, and will further provide a new approach for understanding the structure-sorption relationships of biochars.

## Methods

### Data Collection

To discover the effect of charring temperatures and precursors on H/C atomic ratios, we collected 11 different precursors with 68 data groups from our previous works (the left side in Table S-1), which were prepared using the same procedures (please refer to the preparation procedures in the next section for details). Moreover, 89 data groups from other labs with different preparation procedures and 31 precursors were also collected (the right side in Table S-1).The precursors included biomass (such as shaddock peel, bagasse, rice straw^47^, bamboo wood, pine needle^3^,^62^, orange peels^7^, pine wood^20^, fir wood chips,^25^ rice bran^27^, rice husk^63^, pinewood^64^, poplar wood^65^, spruce wood^65^, wheat straw^65^, cotton seed hull^66^, saw dust^67^, corn stover^42^,^68^, fescue grass^22^, pine wood shavings^22^, buffalo weed^62^, rapeseed plant^43^, peanut shell^69^, soybean stover^69^, oak bark^70^, and corn cobs^68^), manures (such as chicken manure, feed lot^71^, poultry litter^71^, swine solids^71^,^72^, turkey litter^71^, broiler litter^73^, chicken litter^72^, and poultry manure^62^), sludge (such as sewage sludge^62^ and paper sludge^62^) and polymers (such as tire rubber^74^, cellulose^44^ and chitin^44^).

The data connecting the properties and the sorption behavior of biochars were also collected to determine the structure-sorption relationship. Naphthalene (NAP) and phenanthrene (PHE) were selected as the sorbate models for HOCs onto biochars because they have attracted much interest and had the most published data. Because the Freundlich model was the most widely used equation to fit the sorption isotherms, the Freundlich parameters were collected to represent the sorption behaviors of various biochars. Tables S-2 and S-3 listed sorption data of NAP and PHE from a series of biochars, with the reported information including precursor feedstock, preparation methods, heating temperatures, elemental content (C, H, O, N), ash content, H/C atomic ratio, Freundlich parameters (*N* index and log *K*_f_), log *Q*_e_ (*C*_e_ = 0.01*C*_s_), log *Q*_e_ (*C*_e_ = 0.1*C*_s_) and log *Q*_A_ (the logarithmic maximum adsorption capacity). In total, 13 different precursors and 65 data groups were collected for NAP sorption to biochars (Table S-2) and 40 different types of precursors and 98 data groups were collected for PHE sorption to biochars (Table S-3). All of the log *K*_f_ values were ash-corrected after the unit transformation to represent organic matter-normalized log *K*_f_ because the ash content in biochar generally contributed much less to the sorption process, especially for NAP and PHE. log*Q*_e_ values at *C*_e_ = 0.01 *C*_s_ and *C*_e_ = 0.1 *C*_s_ were calculated using the linear form of the Freundlich equation (log *Q*_e_ = log*K*_f_ + *N*log*C*_e_), where *C*_e_ represented the equilibrium sorbate concentration in water and *C*_s_ represented the water solubility in water of the sorbate.

### Biochar preparation procedures and elemental analysis

The preparation procedures of biochar in our lab were generally conducted under the same oxygen-limited conditions as described in previous reports^3^,^7^,^20^,^25^,^27^. To be more specific, the precursors were first washed and air dried for approximately two days. After passing through a 0.154 mm sieve for more uniform feedstock, they were stored in ceramic pots and put into a muffle furnace. The pots were then heated at a rate of 5 °C/min, held for 6 hours at a specific pyrolysis temperature, and then cooled down to room temperature naturally. The biochar samples were obtained after passing through a 0.154 mm sieve. Element analysis (C, H, N) was conducted using the same instrument (EA 112 CHN elemental analyzer, Thermo Finnigan). The oxygen content was generally calculated with the assumption that all organic matter in biochars consisted of C,H,O and N after the ash correction.

### Processing of collected data

The Freundlich isotherm was widely used to fit the sorption isotherm data, and the parameters were commonly obtained by applying its linear form, written as follows:


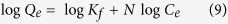


Where *Q*_e_ was the equilibrium adsorbed quantity per mass of sorbent, mg kg^−1^; *C*_e_ was the equilibrium concentration, mg L^−1^; *N* (dimensionless) and *K*_f,_ (mg kg^−1^) (L mg^−1^)^N^ were the Freundlich constants. To make the sorption capacity of different biochars comparable, all of the log *K*_f_ values were ash-corrected to normalize them to the organic matter content in biochar as the ash content generally contributed much less to the sorption than the organic matter for HOCs on biochars. The values of log *Q*_e_ were calculated using the linear form of the Freundlich equation at *C*_e_ = 0.01 *C*_s_ and *C*_e_ = 0.1 *C*_s_ to represent the total sorption capacity at the same *C*_e_ value to make them comparable. To develop the structure-sorption relationship of biochars, the maximum adsorption capacity was valuable for assessing the adsorption capacity of biochars because the partition mechanism was similar to the dissolving pattern, which was limited by the sorbate solubility in water. In the previous study, Chen *et al*.^3^ achieved a good Freundlich fitting result on the sorption isotherm of HOCs onto biochars, and distinguished the contributions of partitioning and adsorption to the total sorption using a high-concentration linear fitting method. Therefore, the Freundlich equation actually represented a combination of both partitioning and adsorption mechanisms. By this isotherm-separation method, the maximum adsorption capacity was achieved from the high-concentration linear fitting. Considering that many published studies did not calculate the maximum adsorption capacity but instead offered the corresponding fitted Freundlich isotherm parameters (as listed in Table S-2, Table S-3), we decided to approximately calculate the maximum adsorption capacity from Freundlich parameters using the high-concentration linear fitting method. Figure S-1 provides a graphical illustration of this approximated calculation. Two points were used for the high-concentration linear fitting from the Freundlich isotherm, at 0.75C_s_ and *C*_s_. The maximum adsorption capacity *Q*_A_ (mg kg^−1^) was equal to *K*_f_*C_s_^*N*^ − 4*(*K*_f_*C_s_^*N*^ − *K*_f_*(0.75 *C*_s_)^*N*^), where *C*_s_(mg L^−1^) represents the sorbate solubility in water.

## Additional Information

**How to cite this article**: Xiao, X. *et al*. H/C atomic ratio as a smart linkage between pyrolytic temperatures, aromatic clusters and sorption properties of biochars derived from diverse precursory materials. *Sci. Rep.* **6**, 22644; doi: 10.1038/srep22644 (2016).

## Supplementary Material

Supplementary Information

## Figures and Tables

**Figure 1:**
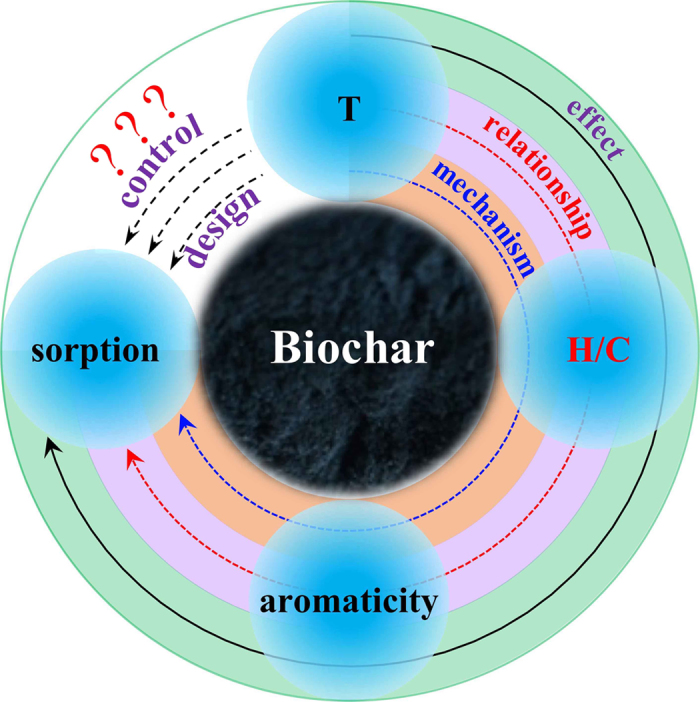
The scheme of the current study on biochar sorption to establish the structure-property relationships between the charring temperature (T), H/C atomic ratio, aromatic cluster and sorption of various biochars. The knowledge to link these parameters need to be shifted from a qualitative effect to a quantitative relationship, and then to an intrinsic mechanism, which is important for controlling and designing specific characteristics of various biochars.

**Table 1:** The relationship between n*n and the H/C atomic ratio according to the rectangle-like polycyclic aromatic ring model (set *m* = *n*).

n*n	**H/C ratio**	n*n	**H/C ratio**
1*1	1.0000	14*14	0.1295
2*2	0.6250	15*15	0.1216
3*3	0.4667	16*16	0.1146
4*4	0.3750	17*17	0.1084
5*5	0.3143	18*18	0.1028
6*6	0.2708	19*19	0.0977
7*7	0.2381	20*20	0.0932
8*8	0.2125	50*50	0.0388
9*9	0.1919	100*100	0.0197
10*10	0.1750	500*500	0.0040
11*11	0.1608	1000*1000	0.0020
12*12	0.1488	10000*10000	0.0002
13*13	0.1385		
