# Supplementary material for: H/C atomic ratio as a smart linkage between pyrolytic temperatures, aromatic clusters and sorption properties of biochars derived from diverse precursory materials
Source: Sci Rep. 2016 Mar 4;6:22644. doi: 10.1038/srep22644 (PMC4778134; doi:10.1038/srep22644)
Supplement: Supplementary Information [file srep22644-s1.pdf]

Appendix A. Supplementary data for *Scientific Reports*

H/C Atomic Ratio as a Smart Linkage between Pyrolytic Temperatures, Aromatic Clusters and Sorption Properties of Biochars Derived from Diverse Precursory Materials

Xin Xiao[1,2], Zaiming Chen[1,3] & Baoliang Chen[1,2*]

1. Department of Environmental Science, Zhejiang University, Hangzhou 310058, China;

2. Zhejiang Provincial Key Laboratory of Organic Pollution Process and Control, Hangzhou 310058, China;

3. Department of Environmental Engineering, Ningbo University, Ningbo 315211, China.

*Corresponding Author

Phone: 0086-571-88982587; fax: 0086-571-88982587; e-mail: blchen@zju.edu.cn.

Supporting Information consists of 23 pages, including this one.

There are 5 Tables and 2 Figures.

Table S-1 The data of pyrolysis temperature and H/C atomic ratio from various precursor substances. The left side shows the results from our group, and the right side from others.

precursor substances	pyrolysis temperature (º C)	H/C atomic ratio	precursor substances	pyrolysis temperature (º C)	H/C atomic ratio
**shaddock peel** [a]	700	0.16	rice husk[1] [b]	500	0.63
			pinewood[2] (2h)	700	0.10
**chicken manure**	100	1.75	poplar wood[3] (10h)	400	0.79
	150	1.64		460	0.60
	200	1.37		525	0.41
	250	1.12	spruce wood[3] (10h)	400	1.04
	300	0.90		460	0.50
	400	0.60		525	0.47
	450	0.49	wheat straw[3] (5h)	400	0.74
	550	0.32		460	0.52
	600	0.29		525	0.46
**bagasse**	100	1.70	feed lot[4] (2h)	350	0.91
	200	1.21		700	0.21
	300	0.70	poultry litter[4] (2h)	350	0.89
	400	0.51		700	0.52
	500	0.39	swine solid[4] (2h)	350	1.14
	600	0.38		700	0.20
	700	0.23	turkey litter[4] (2h)	350	0.88
				700	0.24
**rice straw**	25	1.64	cotton seed hull[5] (4h) [c]	25	1.50
	150	1.56		200	1.38
	250	1.11		350	0.70
	350	0.72		500	0.39
	500	0.36		650	0.17
	700	0.14		800	0.08
**bamboo wood**	100	1.54	broiler litter[6] (1h)	350	1.05
	200	1.31		700	0.37
	300	0.70	saw dust[7]	450	0.58
	400	0.52		550	0.14

	500	0.39	chicken litter[8] (2h)	620	0.35
	600	0.26	swine solid[8] (2h)	620	0.45
	700	0.20	corn stover[9]	450	0.51
**pine needle[10]**	100	1.44	fescue grass[11] (1h)	100	1.59
	200	1.19		200	1.64
	250	1.08		300	1.42
	300	0.75		400	0.80
	400	0.45		500	0.52
	500	0.33		600	0.40
	600	0.26		700	0.21
	700	0.18	pine wood shaving[11] (1h)	100	1.81
				200	1.81
				300	1.34
				400	0.73
				500	0.48
				600	0.33
				700	0.20
**rice straw[12]**	100	1.72	poultry manure[13]	300	0.89
	200	1.30		400	0.74
	300	0.76		500	0.44
	400	0.58		600	0.33
	500	0.49		700	0.32
	600	0.36	buffalo weed[13]	300	0.65
	700	0.32		700	0.15
			sewage sludge[13]	300	1.21
				400	0.87
				500	0.64
				600	0.40
				700	0.31
			paper sludge[13]	105	1.48
				300	0.74
				700	0.14
			pine needles[13]	300	0.62
				500	0.27

				700	0.08
**orange peel[14]**	150	1.46	rapeseed plant[15] (0.5h)	400	0.66
	200	1.14		500	0.42
	250	0.94		600	0.28
	300	0.78		700	0.18
	350	0.68		800	0.11
	400	0.58		900	0.06
	500	0.38	tire rubber[16] (2h)	200	1.02
	600	0.30		400	0.55
	700	0.29		600	0.25
				800	0.12
**pine wood[17]**	150	1.51	peanut shell[18] (3h)	300	0.68
	250	0.90		700	0.25
	350	0.47	soybean stover[18] (3h)	300	0.75
	500	0.38		700	0.19
	700	0.14	oak bark[19]	450	0.44
**fir wood chip[20]**	200	1.44	cellulose[21] (8h)	25	1.92
	300	0.69		250	0.47
	400	0.52		320	0.51
	500	0.37		400	0.50
	600	0.25	chitin[21] (8h)	25	1.84
	700	0.14		250	0.58
				320	0.56
				400	0.49
**rice bran[22]**	100	1.71	corn cob[23]	500	0.47
	300	0.75	corn stover[23]	500	0.60
	700	0.20			

[a]: The biochars in the left side were all prepared by the heating rate of 5 °C min^{-1} and a holding time of 6h.
[b]: The biochars in the right side were prepared by different preparing method. The holding time period was provided in the brackets behind the precursors. The precursors with no preparing parameter (such as holding period) mentioned were not followed by bracket.

Table S-2 The sorption parameters of naphthalene onto a series of biochars (including 13 biomass data)

precursor substances	preparing methods	heating temperature (°C)	C (wt.%)	H (wt.%)	O (wt.%)	N (wt.%)	ash (wt.%)	H/C	N	log K_f [a]	log Q_e (C_e=0.01C_s) [b]	log Q_e (C_e=0.1C_s) [b]	log Q_A [c]
pine needle[10]	5 ° C min^{-1} [d], 6h [e], HCl treated	100	50.34	6.09	41.82	0.7	1.05	1.440	1.107	2.347	1.799	2.906	
		200	56.58	5.66	36.31	0.87	0.9	1.191	0.874	3.040	2.607	3.481	3.383
		250	60.48	5.47	32.36	0.85	1.24	1.077	0.777	3.217	2.833	3.610	3.677
		300	67.55	4.23	25.74	1.06	1.91	0.746	0.589	3.502	3.211	3.800	3.962
		400	76.04	2.88	18.04	1.13	2.32	0.451	0.332	4.232	4.068	4.400	4.534
		500	79.38	2.2	14.96	1.08	2.8	0.329	0.207	4.298	4.196	4.403	4.495
		600	83	1.8	11.81	0.95	2.76	0.258	0.174	4.109	4.023	4.197	4.276
		700	84.61	1.25	11.08	1.1	2.2	0.176	0.124	5.095	5.033	5.157	5.215
orange peels[14]	5° C min^{-1}, 6h, HCl treated	150	50.6	6.2	41	1.75	0.45	1.470	1.098	2.422	1.879	2.977	
		200	57.9	5.53	34.39	1.88	0.3	1.146	0.771	3.231	2.850	3.621	3.694
		250	65.1	5.12	26.51	2.22	1.05	0.944	0.815	3.485	3.081	3.896	3.916
		300	69.3	4.51	22.26	2.36	1.57	0.781	0.683	3.667	3.329	4.012	4.147
		350	73.2	4.19	18.31	2.3	2	0.687	0.537	3.779	3.513	4.050	4.215
		400	71.7	3.48	20.8	1.92	2.1	0.582	0.516	3.839	3.584	4.100	4.265
		500	71.4	2.25	20.25	1.83	4.27	0.378	0.384	3.269	3.079	3.463	
		600	77.8	1.97	14.39	1.8	4.04	0.304	0.249	3.168	3.045	3.294	
		700	71.6	1.76	22.13	1.72	2.79	0.295	0.151	4.782	4.708	4.859	4.928
pine wood[17]	5° C min^{-1}, 6h, HCl treated	150	49.2	6.2	43.6	0.26	1.04	1.512	0.947	2.723	2.254	3.201	2.768
		250	57.7	4.33	36.3	0.275	1.74	0.901	0.667	3.614	3.284	3.951	4.092
		350	65.4	2.57	29.8	0.352	2.26	0.472	0.451	4.163	3.940	4.391	4.550
		500	69.7	2.18	23.8	0.384	4.32	0.375	0.209	4.787	4.684	4.893	4.986
		700	84.9	0.987	9.36	0	4.74	0.140	0.111	5.227	5.172	5.283	5.336
rice straw[12]	5° C min^{-1}, 6h, HCl treated	300	42.09	2.66	17.06	1.19	37	0.758	0.644	4.100	3.781	4.425	4.575
cellulose[21]	8h	25	41.4	6.62	51.98	0	0	1.919	0.992	0.891	0.400	1.392	1.000
		250	64.9	2.52	32.58	0	0	0.466	0.256	4.516	4.389	4.645	4.756

		320	65.3	2.8	31.9	0	0	0.515	0.327	4.643	4.481	4.808	4.941
		400	77.2	3.24	19.56	0	0	0.504	0.407	4.722	4.521	4.928	5.080
chitin[21]	8h	25	43.8	6.72	43.03	6.45	0	1.841	1.008	0.972	0.473	1.481	
		250	55.6	2.69	30.51	11.2	0	0.581	0.426	4.088	3.877	4.303	4.459
		320	55.6	2.61	27.49	14.3	0	0.563	0.437	4.154	3.938	4.375	4.532
		400	57.1	2.33	25.47	15.1	0	0.490	0.441	4.236	4.018	4.459	4.617
a-amylase[24]	8h	170	43.3	5.69	44.01	7	0	1.577	0.989	2.797	2.308	3.297	1.973
		250	50	4.81	37.67	7.52	0	1.154	0.834	3.482	3.069	3.903	3.892
		350	48.7	3.93	39.85	7.52	0	0.968	0.659	3.765	3.439	4.098	4.243
		450	51.3	1.88	40.06	6.76	0	0.440	0.515	3.627	3.372	3.887	4.052
chitin[24]	8h	170	43.9	6.67	42.97	6.46	0	1.823	0.846	1.293	0.874	1.720	1.687
		250	47	6.18	39.63	7.19	0	1.578	0.849	2.438	2.018	2.867	2.827
		350	72.3	3.94	13.17	10.59	0	0.654	0.623	4.087	3.779	4.402	4.557
		450	67	2.98	17.8	12.22	0	0.534	0.562	3.823	3.545	4.107	4.271
zein[24]	8h	170	52.8	7.34	24.9	14.96	0	1.668	0.996	2.485	1.992	2.988	
		250	62.7	6.69	15.77	14.84	0	1.280	0.905	3.103	2.655	3.560	3.362
		350	61.8	4.06	21.59	12.55	0	0.788	0.685	3.622	3.283	3.968	4.102
		450	68.9	3.27	14.71	13.12	0	0.570	0.649	4.520	4.199	4.848	4.996
maple wood shavings[25]	25° C min^{-1}, 2h	300	60.81	5.62	31.06	<0.5	0.81	1.109	0.76	3.794	3.417	4.177	4.261
		350	72.53	4.63	21.69	<0.5	0.61	0.766	0.77	3.973	3.592	4.362	4.436
		400	74.78	4.01	18.56	<0.5	1.66	0.643	0.66	3.727	3.401	4.061	4.205
		500	83.16	3.24	10.59	<0.5	1.79	0.468	0.21	4.248	4.144	4.354	4.447
		600	86.31	2.52	7.15	<0.5	2.2	0.350	0.21	3.810	3.706	3.916	4.009
		700	84.43	1.86	6.7	<0.5	3.37	0.264	0.35	3.055	2.882	3.232	
maize stalk[26]	3h	300	66.79	4.92	19.25	1.88	7.15	0.884	0.73	3.242 [f]	2.880	3.610	3.719
		500	72.12	3.18	14.15	--	10.56	0.529	0.89	3.377 [f]	2.936	3.826	3.682
		700	76.3	3.38	9.53	--	10.78	0.532	0.33	3.912 [f]	3.748	4.078	4.212
bagasse	5 ° C min^{-1}, 6h HCl treated	200	53.71	5.42	39.57	0.88	0.42	1.211	0.95	2.705	2.235	3.185	2.726
		300	68.4	4.01	24.62	1.61	1.36	0.704	0.593	3.619	3.326	3.919	4.080

		400	74.12	3.15	19.86	1.27	1.6	0.510	0.317	4.040	3.883	4.200	4.330
		500	79.47	2.58	14.63	1.47	1.85	0.390	0.306	4.514	4.363	4.669	4.795
		600	79.55	2.49	14.18	1.59	2.19	0.376	0.358	4.569	4.391	4.749	4.891
		700	81.79	1.57	13.16	1.51	1.97	0.230	0.21	5.186	5.082	5.292	5.385
bamboo	5° C min^{-1}, 6h	100	47.92	6.16	44.66	0.24	1.02	1.543	0.94	2.235 [f]	1.770	2.710	2.329
		200	52.64	5.74	40.22	0.34	1.06	1.309	0.81	3.209 [f]	2.808	3.618	3.645
		300	70.72	4.15	22.39	0.62	2.12	0.704	0.59	3.800 [f]	3.508	4.098	4.260
		400	77.09	3.32	15.75	0.69	3.15	0.517	0.34	4.146 [f]	3.977	4.317	4.454
		500	83.01	2.67	10	0.63	3.69	0.386	0.21	4.504 [f]	4.400	4.610	4.704
		600	84.48	1.84	9.64	0.52	3.52	0.261	0.08	4.903 [f]	4.863	4.943	4.981
		700	86.83	1.42	8.02	0.45	3.28	0.196	0.07	5.237 [f]	5.202	5.272	

[a]: Ash corrected. The values of log K_f are all ash corrected. The equation that is used for ash correcting is written as log K_f = log K_f' – log (1-η), where K_f' was the published data after unit transformation and η was the ash content (wt.%) in biochars. K_f, K_f': (mg kg^{-1}) (L mg^{-1})^N

[b]: The calculated log Q_e using the linear Freundlich equation at C_e = 0.01 C_s (or C_e=0.1C_s), where C_e represented the equilibrium sorbate concentration in water, and C_s represented the water solubility in water of the sorbate. The linear Freundlich equation was written as: $\log Q_e = \log K_f + N \log C_e$

[c]: log Q_A represents the logarithmic maximum adsorption capacity generated from the high-concentration linear fitting of two points in the Freundlich equation at 3/4 C_s and C_s equilibrium concentration. C_s refers to the naphthalene solubility in water that was 32 mg L^{-1} according to reference [10].

[d]: The unit ° C min^{-1} refers to the heating rate.

[e]: The time period listed behind (such as 3h, 6h, 8h etc.) refers to the holding time.

[f]: Unit transformation: The log K_f' was obtained from the equation (log K_f' = log K_f^0+3) due to the unit transformation, where K_f^0 was the published data with unit of (mg g^{-1}) (L mg^{-1})^N in the article.

Table S-3 The sorption parameters of phenanthrene onto a series of biochars (including 7 biomass data).

precursor substances	preparing methods	heating temperature (°C)	C (wt.%)	H (wt.%)	O (wt.%)	N (wt.%)	ash (wt.%)	H/C	*N*	log K_f [a]	log Q_e (C_e=0.01C_s) [b]	log Q_e (C_e=0.1C_s) [b]	log Q_A [c]
pine needle[10,27]	5 ° C min^{-1} [d], 6h [e], HCl treated	100	50.34	6.09	41.82	0.7	1.05	1.452	1.001	3.985	2.043	3.044	1.000
		300	67.55	4.23	25.74	1.06	1.91	0.751	0.605	3.780	2.607	3.212	3.375
		400	76.04	2.88	18.04	1.13	2.32	0.454	0.449	4.355	3.484	3.933	4.095
		700	84.61	1.25	11.08	1.1	2.2	0.177	0.428	5.134	4.304	4.732	4.889
rice straw[12]	5° C min^{-1}, 6h, HCl treated	300	66.84	4.22	17.06	1.88	37	0.758	0.855	5.080	3.422	4.277	4.238
rice straw[28]	10 ° C min^{-1}, 1h, HCl treated	450	57.9	3.31	11.8	0.83	26.2	0.686	0.43	4.152	3.318	3.748	3.906
		600	59.3	2.34	5.5	0.83	32	0.474	0.38	4.427	3.691	4.071	4.218
	10 ° C min^{-1}, 1h, HCl+HF treated	450(deash)	72.5	3.71	15.4	1.17	7.2	0.614	0.5	4.592	3.623	4.123	4.289
		600(deash)	80.4	2.53	7.4	1.14	8.6	0.378	0.4	4.779	4.003	4.403	4.556
wheat straw[28]	10° C min^{-1}, 1h, HCl treated	450	70.2	4.28	12.9	0.46	12.2	0.732	0.48	4.167	3.236	3.716	3.881
		600	77.8	3.08	5.3	0.42	13.4	0.475	0.41	4.162	3.367	3.777	3.932
	10° C min^{-1}, 1h, HCl+HF treated	450(deash)	74.1	3.51	15.2	0.66	6.6	0.568	0.47	4.410	3.498	3.968	4.132
		600(deash)	83.9	2.65	5.9	0.45	7.2	0.379	0.49	4.422	3.472	3.962	4.128
maize stalk[28]	10° C min^{-1}, 1h, HCl treated	450	74.4	3.81	11.8	1.01	9.1	0.615	0.43	3.971	3.138	3.568	3.726
		600	79.9	3.71	5.6	0.99	9	0.557	0.54	4.771	3.724	4.264	4.432
	10° C min^{-1}, 1h, HCl+HF treated	450(deash)	78.8	3.31	14	1.29	2.6	0.504	0.55	4.391	3.325	3.875	4.042
		600(deash)	84.2	2.36	6.9	1.16	5.4	0.336	0.46	4.614	3.722	4.182	4.345
chicken manures[28]	10° C min^{-1}, 1h, HCl treated	450	9.8	0.91	3.6	0.53	85.2	1.114	0.49	4.360	3.409	3.899	4.065
		600	8.7	0.5	1.5	0.33	89	0.690	0.42	4.529	3.714	4.134	4.290
	10° C min^{-1}, 1h, HCl+HF treated	450(deash)	26.1	2.24	15.9	1.4	54.5	1.030	0.48	4.642	3.711	4.191	4.356
		600(deash)	22.6	1.42	12	0.97	63.1	0.754	0.7	5.093	3.735	4.435	4.567
swine manures[28]	10° C min^{-1}, 1h,	450	33.7	2.55	10.2	2.57	50.9	0.908	0.48	4.289	3.358	3.838	4.003

	HCl treated	600	35.6	1.79	7.9	2.46	52.3	0.603	0.29	4.481	3.919	4.209	4.331
	10° C min^{-1}, 1h, HCl+HF treated	450(deash)	43.4	2.77	9.9	3.53	40.4	0.766	0.54	4.915	3.868	4.408	4.575
		600(deash)	42.8	2.44	6.8	2.76	45.2	0.684	0.44	4.791	3.938	4.378	4.538
cow manures[28]	10° C min^{-1}, 1h, HCl treated	450	29.5	0.95	4.1	1.39	68.1	0.386	0.55	4.546	3.480	4.030	4.197
		600	30.7	0.46	1.2	1.11	71.2	0.180	0.35	4.411	3.732	4.082	4.222
	10° C min^{-1}, 1h, HCl+HF treated	450(deash)	51.4	2.76	14.6	2.25	28.9	0.644	0.53	4.938	3.910	4.440	4.608
		600(deash)	50.5	1.93	10	1.9	35.7	0.459	0.4	4.792	4.016	4.416	4.568
cellulose[21]	8h	25	41.4	6.62	51.98	0	0	1.919	1.068	2.239	0.168	1.236	1.000
		250	64.9	2.52	32.58	0	0	0.466	0.46	4.194	3.302	3.762	3.925
		320	65.3	2.8	31.9	0	0	0.515	0.508	4.778	3.793	4.301	4.468
		400	77.2	3.24	19.56	0	0	0.504	0.543	4.981	3.928	4.471	4.639
chitin[21]	8h	25	43.8	6.72	43.03	6.45	0	1.841	1.015	2.241	0.273	1.288	1.000
		250	55.6	2.69	30.51	11.2	0	0.581	0.515	4.570	3.571	4.086	4.254
		320	55.6	2.61	27.49	14.3	0	0.563	0.511	4.677	3.686	4.197	4.364
		400	57.1	2.33	25.47	15.1	0	0.490	0.536	4.815	3.776	4.312	4.479
a-amylase[24]	8h	170	43.3	5.69	44.01	7	0	1.577	1.038	3.972	1.959	2.997	1.000
		250	50	4.81	37.67	7.52	0	1.154	0.824	4.770	3.172	3.996	4.013
		350	48.7	3.93	39.85	7.52	0	0.968	0.783	4.807	3.289	4.072	4.141
		450	51.3	1.88	40.06	6.76	0	0.440	0.475	4.037	3.116	3.591	3.755
chitin[24]	8h	170	43.9	6.67	42.97	6.46	0	1.823	0.864	2.016	0.340	1.204	1.147
		250	47	6.18	39.63	7.19	0	1.578	0.814	3.273	1.694	2.508	2.540
		350	72.3	3.94	13.17	10.59	0	0.654	0.576	4.511	3.394	3.970	4.136
		450	67	2.98	17.8	12.22	0	0.534	0.543	4.175	3.122	3.665	3.833
zein[24]	8h	170	52.8	7.34	24.9	14.96	0	1.668	0.986	3.509	1.597	2.583	1.652
		250	62.7	6.69	15.77	14.84	0	1.280	0.984	4.240	2.332	3.316	2.441
		350	61.8	4.06	21.59	12.55	0	0.788	0.797	4.045	2.499	3.296	3.350
		450	68.9	3.27	14.71	13.12	0	0.570	0.564	4.158	3.064	3.628	3.795
malt spent rootlets (MSR)[29]	1.5h	300	53	2.8		4.5		0.634	0.55	3.230 [f]	2.163	2.713	2.881

MSR-MeOH[29]		300	55	3.4		3.6		0.742	0.51	2.910 [f]	1.921	2.431	2.598
MSR-NaOH [29]		300	59	3.9		4.8		0.793	0.43	2.892 [f]	2.058	2.488	2.646
cotton straw[30]	10° C min^{-1}, 1h, HCl treated	300	67.1	4.6	20.7	1.3	6.3	0.823	0.519	3.445 [g]	2.439	2.958	3.125
potato straw[30]		300	59.1	5.2	19.7	4	12.1	1.056	0.657	3.877 [g]	2.603	3.260	3.410
leaf[30]		300	62.2	5.4	16.9	4.8	10.7	1.042	0.63	3.839 [g]	2.617	3.247	3.405
rice straw[30]		300	55.3	3.7	19.5	0.8	20.6	0.803	0.513	3.669 [g]	2.674	3.187	3.355
wheat straw[30]		300	63.3	4.4	24	0.5	7.8	0.834	0.588	3.589 [g]	2.449	3.037	3.202
maize straw[30]		300	63.8	5.1	25.3	0.8	5.1	0.959	0.506	3.271 [g]	2.289	2.795	2.962
nut[30]		300	60.9	5.4	29.2	0.2	4.3	1.064	0.827	3.220 [g]	1.616	2.443	2.455
wood dust[30]		300	65.4	5.3	27.3	0	2	0.972	0.496	3.097 [g]	2.135	2.631	2.797
chicken manures[30]		300	10	0.9	4.6	0.6	83.9	1.080	0.531	4.046 [g]	3.016	3.547	3.715
swine manures[30]		300	36.5	3.6	14.9	3.2	41.8	1.184	0.519	3.672 [g]	2.666	3.185	3.352
cotton straw[30]		450	71.6	3.9	13.3	1.2	10.1	0.654	0.717	3.667 [g]	2.277	2.994	3.116
potato straw[30]		450	61.9	3.7	14.3	3.7	16.4	0.717	0.72	4.038 [g]	2.641	3.361	3.482
leaf[30]		450	63.4	3.7	12.6	5	15.2	0.700	0.778	3.866 [g]	2.357	3.135	3.209
rice straw[30]		450	57.9	3.3	11.8	0.8	26.2	0.684	0.727	3.783 [g]	2.373	3.100	3.216
wheat straw[30]		450	70.2	4.3	12.9	0.5	12.2	0.735	0.757	3.758 [g]	2.289	3.046	3.140
maize straw[30]		450	74.4	3.8	11.8	1	9.1	0.613	0.689	3.658 [g]	2.322	3.011	3.149
nut[30]		450	78.4	3.6	11.8	0.3	6	0.551	0.845	3.152 [g]	1.513	2.358	2.339
wood dust[30]		450	75.9	3.7	16.7	0.1	3.7	0.585	0.787	3.657 [g]	2.131	2.918	2.983
chicken manures[30]		450	9.8	0.9	3.6	0.5	85.2	1.102	0.897	4.211 [g]	2.471	3.368	3.221
swine manures[30]		450	33.7	2.6	10.2	2.6	50.9	0.926	0.732	3.905 [g]	2.485	3.217	3.330
rice straw[31]	1h, HCl treated	300	53.2	3.9	24.2	1.1	17.6	0.880	0.55	4.214 [g]	3.147	3.697	3.865
		450	57	2.6	15.6	1.2	23.6	0.547	0.45	4.137 [g]	3.264	3.714	3.875
		600	60.4	1.7	8.9	1.1	27.9	0.338	0.38	4.402 [g]	3.665	4.045	4.193
pine wood[31]	1h, HCl treated	300	64.7	4.8	28.6	0	1.9	0.890	0.63	3.828 [g]	2.607	3.237	3.394
		450	73.1	2.8	20.1	0.1	3.9	0.460	0.54	4.227 [g]	3.180	3.720	3.888

		600	81.4	2.3	11.7	0.1	4.4	0.339	0.71	5.010 [g]	3.633	4.343	4.469
rice straw (bleached)[31]	1h, HCl treated, $NaClO_2$+CH_3C OOH treated.	300	26.9	3	24.9	0.4	44.9	1.338	0.76	3.779 [g]	2.306	3.066	3.157
		450	39.2	2.2	27.8	0.7	30.1	0.673	0.55	4.048 [g]	2.981	3.531	3.699
		600	50.2	1.6	19.6	0.8	27.8	0.382	0.25	3.299 [g]	2.814	3.064	3.173
pine wood (bleached)[31]	1h, HCl treated, $NaClO_2$+CH_3C OOH treated.	300	43.1	5	45.4	0.1	6.4	1.392	0.75	3.694 [g]	2.239	2.989	3.089
		450	51.6	2.4	36.6	0	9.4	0.558	0.61	4.058 [g]	2.875	3.485	3.646
		600	65.1	2.2	25.8	0	6.8	0.406	0.89	5.052 [g]	3.326	4.216	4.090
cotton[32]	2h, HCl treated	450	71.6	3.89	13.3	1.17	10.1	0.652	0.41	4.016 [g]	3.221	3.631	3.785
soybean[32]		450	70.8	3.92	15.6	0.98	8.7	0.664	0.48	4.180 [g]	3.249	3.729	3.894
rice[32]		450	57.9	3.31	11.8	0.83	26.2	0.686	0.45	4.152 [g]	3.279	3.729	3.890
wood dust[32]		450	75.9	3.66	16.7	0.05	3.7	0.579	0.54	4.226 [g]	3.179	3.719	3.887
swine waste[32]		450	33.7	2.55	10.2	2.57	50.9	0.908	0.49	4.279 [g]	3.329	3.819	3.985
Dianchi lake sediment[33,34]	4h	0	22.04	2.42	26.04	1.52	47.98	1.318	0.978	3.834	1.937	2.915	2.173
		200	23.85	1.36	23.34	1.74	49.71	0.684	0.865	4.149	2.471	3.336	3.276
		300	21.93	1.06	20.71	1.29	55.01	0.580	0.713	4.617	3.234	3.947	4.072
		400	20.26	0.59	18.56	1.08	59.51	0.349	0.566	4.683	3.585	4.151	4.318
		500	20.43	0.4	18.23	1.07	59.87	0.235	0.325	4.277	3.646	3.971	4.104
poultry litter （hydrothermal)[35]	20h	250	47.46	5.72	20.7	1.25	24.9	1.446	0.91	4.724 [g]	2.960	3.870	3.676
swine soild (hydrothermal)[35]	20h	250	40.2	3.86	22.1	1.67	32.2	1.152	0.75	4.619 [g]	3.164	3.914	4.014
poultry litter （thermal)[35]	120–420 min, HCl treated	400	53.45	3.71	15	2.8	25	0.833	0.67	4.585 [g]	3.286	3.956	4.101
wheat straw （thermal)[35]	120–420 min, HCl treated	400	65.79	3.43	20.4	0.21	10.2	0.626	0.6	3.927 [g]	2.763	3.363	3.526

[a]: ash corrected. The values of log K_f are all ash corrected. The equation that is used for ash correcting is written as log K_f = log K_f' – log (1-η), where K_f' was the published data after unit transformation and η was the ash content (wt.%) in biochars. K_f, K_f': (mg kg^{-1}) (L mg^{-1})^N

[b]: the calculated log Q_e using the linear Freundlich equation at C_e = 0.01 C_s (or C_e=0.1C_s), where C_e represented the equilibrium sorbate concentration in water, and C_s represented the water solubility in water of the sorbate. The linear Freundlich equation was written as: $\log Q_e = \log K_f + N \log C_e$

[c]: log Q_A represents the logarithmic maximum adsorption capacity generated from the high-concentration linear fitting of two points in the Freundlich equation at 3/4 C_s and C_s equilibrium concentration. C_s refers to the phenanthrene solubility in water that was 1.15mg L^{-1} according to reference[24].
[d]: The unit ° C min^{-1} refers to the heating rate.
[e]: The time period listed behind (such as 3h, 6h, 8h etc.) refers to the holding time.
[f]: Unit transformation: The log $K_f^{'}$ was obtained from the equation (log $K_f^{'}$ = log K_f^0+3*N-3) due to the unit transformation, where K_f^0 was the published data with unit of (μg kg^{-1}) (L $μg^{-1})^N$ in the article.
[g]: Unit transformation: The log $K_f^{'}$ was obtained from the equation (log $K_f^{'}$ = log K_f^0+3*N) due to the unit transformation, where K_f^0 was the published data with unit of (μg g^{-1}) (L $μg^{-1})^N$ in the article.

Table S-4 The fitted results between H/C ratio and the sorption parameters for naphthalene (NAP) and phenanthrene (PHE) to diverse biochars. Please refer to Tables S-2 and S-3 for specific data, and refer to Figure 3 for the linear fitted figure.

parameters	NAP	PHE
$\boldsymbol{N}$	$N = 0.5551 \times \frac{H}{C} + 0.1329$, ($R^2$=0.8083)	$N = 0.3459 \times \frac{H}{C} + 0.3401$, ($R^2$=0.4727)
**log $\boldsymbol{K_f}$**	$log\ K_f = -1.7363 \times \frac{H}{C} + 5.0205$, ($R^2$=0.7631)	$log\ K_f = -0.8900 \times \frac{H}{C} + 4.8089$, ($R^2$=0.2734)
**log $\boldsymbol{Q_e}$ ($\boldsymbol{C_e}$=0.01$\boldsymbol{C_s}$)**	$log\ Q_e = -2.0110 \times \frac{H}{C} + 4.9548$, ($R^2$=0.8020)	$log\ Q_e = -1.5609 \times \frac{H}{C} + 4.1494$, ($R^2$=0.5139)
**log $\boldsymbol{Q_e}$ ($\boldsymbol{C_e}$=0.1$\boldsymbol{C_s}$)**	$log\ Q_e = -1.4559 \times \frac{H}{C} + 5.0876$, ($R^2$=0.6904)	$log\ Q_e = -1.2149 \times \frac{H}{C} + 4.4895$, ($R^2$=0.4166)
**log $\boldsymbol{Q_A}$**	$log\ Q_A = -1.8664 \times \frac{H}{C} + 5.503$, ($R^2$=0.8225)	$log\ Q_A = -1.7173 \times \frac{H}{C} + 4.9249$, ($R^2$=0.5389)

Table S-5 The sorption parameters of naphthalene and phenanthrene onto other carbon materials (not biochar).

sorbates	samples	C (wt.%)	H (wt.%)	O (wt.%)	N (wt.%)	ash (wt.%)	H/C	N	log K_f [a]	log Q_A (exp) [b]	log Q_A (pre) [c]
naphthalene	humic acid[36]	53.7	4.73	38.8	2.31	0.49	1.057	0.953	2.350	2.346	3.530
		52.2	4.14	40.2	2.85	0.26	0.952	0.969	2.367	2.174	3.727
		50.3	4.38	41.3	2.99	0.36	1.045	0.969	2.382	2.189	3.553
		54.3	3.59	38.3	2.88	0.51	0.793	0.916	2.345	2.563	4.022
		56.8	3.04	36.2	3.51	0.63	0.642	0.893	2.339	2.636	4.304
		56.5	2.98	35.6	3.47	1.84	0.633	0.87	2.354	2.706	4.322
	carbon nanotubes[37]	99.8	1.04	0.1	0	1.42	0.125	0.443	3.750	4.132	5.270
		85.7	0.37	12.1	0.2	1.62	0.052	0.456	3.701	4.092	5.406
		91.9	0.82	5.4	0.45	1.39	0.107	0.572	3.557	4.010	5.303
		84.3	1.32	10.1	2.42	1.89	0.188	0.489	3.219	3.630	5.152
	graphene[38]	76.2	1.03	17.6	5.17	0	0.162			5.106	5.200
	graphene oxide[38]	46.2	2.46	50.9	0.481	0	0.639			3.418	4.310
phenanthrene	humic acid[36]	53.7	4.73	38.8	2.31	0.49	1.057	0.923	3.802	2.629	3.110
		52.2	4.14	40.2	2.85	0.26	0.952	0.932	3.840	2.604	3.291
		50.3	4.38	41.3	2.99	0.36	1.045	0.926	3.818	2.624	3.130
		54.3	3.59	38.3	2.88	0.51	0.793	0.881	3.845	2.883	3.562
		56.8	3.04	36.2	3.51	0.63	0.642	0.868	4.009	3.096	3.822
		56.5	2.98	35.6	3.47	1.84	0.633	0.861	4.015	3.126	3.838
	carbon nanotubes[37]	99.8	1.04	0.1	0	1.42	0.125	0.254	4.394	4.264	4.710
		85.7	0.37	12.1	0.2	1.62	0.052	0.263	4.349	4.214	4.836
		91.9	0.82	5.4	0.45	1.39	0.107	0.263	4.289	4.154	4.741
		84.3	1.32	10.1	2.42	1.89	0.188	0.587	4.248	3.855	4.602
	graphene[38]	76.2	1.03	17.6	5.17	0	0.162			5.135	4.646
	graphene oxide[38]	46.2	2.46	50.9	0.481	0	0.639			3.771	3.828
	sediment[39]	29.1	2.68	7.95	0.93	59.34	1.105	0.643	4.300 [d]	3.842	3.027

		24.2	2.07	5.67	0.6	67.46	1.026	0.561	4.330 [d]	3.965	3.162
		23.4	1.41	5.46	0.54	69.19	0.723	0.521	4.349 [d]	4.022	3.683
		20.6	1.13	4.58	0.43	73.26	0.658	0.489	4.427 [d]	4.129	3.794
		17	0.83	3.42	0.27	78.48	0.586	0.46	4.700 [d]	4.426	3.919
		12.2	0.54	2.14	0.13	84.99	0.531	0.444	4.918 [d]	4.658	4.013
		8.97	0.34	1.36	0.1	89.23	0.455	0.387	5.034 [d]	4.817	4.144
	humic acid[40]	51.9	4.9	39.4	3.8	0.1	1.133	0.8	3.445 [e]	2.725	2.979
		54.7	4.9	37.5	2.9	0.1	1.075	0.81	3.530 [e]	2.786	3.079
		53.9	5	38.3	2.7	0.1	1.113	0.81	3.463 [e]	2.720	3.013
		48.4	4.7	46.3	0.6	0.1	1.165	0.82	3.539 [e]	2.770	2.924
	lignite[41]	60.4	4.76	23	0	2.58	0.946	0.651	4.570	4.102	3.301
		62.3	4.85	22.7	2.12	2.74	0.934	0.637	4.715	4.264	3.321
		64.3	4.71	20.9	2.05	2.86	0.879	0.629	4.796	4.355	3.415
		65.4	5.5	19.6	1.09	3.04	1.009	0.576	4.931	4.550	3.192
		69.9	5.53	16.3	2.47	3.51	0.949	0.493	4.730	4.428	3.295
		75	4.64	13.3	2.72	3.86	0.742	0.391	4.030	3.810	3.650
		77.9	4.46	10.1	2.7	4.21	0.687	0.304	3.567	3.406	3.745
		80.9	3.67	7	2.66	4.54	0.544	0.24	3.504	3.382	3.990
	coal[42]	33.53	4.25	23.63	0.87	37.72	1.521	0.81	4.266	3.522	2.313
		53.94	4.52	26.3	0.76	14.48	1.006	0.71	4.308	3.756	3.198
		32.35	3.2	11.75	0.59	52.11	1.187	0.65	4.430	3.963	2.886
		34.86	2.43	7.74	0.5	54.47	0.836	0.85	5.462	4.609	3.488
		60.45	4.39	15.59	1.09	18.48	0.871	0.72	4.299	3.731	3.428
		65.54	4.48	19.07	0.73	10.18	0.820	0.71	4.387	3.835	3.516
		74.99	4.92	9.86	1.36	8.87	0.787	0.9	4.460	3.415	3.573
		77	4.66	6	1.36	10.98	0.726	0.81	4.511	3.767	3.678
		77.8	4.36	4.82	1.54	11.48	0.672	0.62	4.703	4.273	3.770
		80.13	4	4.21	1.07	10.59	0.599	0.88	5.489	4.531	3.896
		87.31	3.82	4	0.58	4.29	0.525	1.43	5.969 [f]		4.023

	humic acid[43]	47.9					1.180	0.819	3.437 [f]	2.671	2.898
		43.7					1.310	0.881	3.353 [f]	2.392	2.675
		47.5					1.240	0.902	3.263 [f]	2.208	2.795
		50.2					1.380	0.735	3.676 [f]	3.083	2.555
		51.8					1.070	0.941	3.237 [f]	1.928	3.087
		53.8					0.920	0.98	3.031 [f]		3.345
	humic acid[44]	53.1	4.5		2.8	1.2	1.017	0.902	3.650 [d]	3.919	3.178
		54.8	5.3		2.7	1.3	1.161	0.923	3.868 [d]	2.694	2.932
		55.7	5.7		2.2	1.1	1.228	0.958	3.947 [d]	2.453	2.816
		57.7	6.2		2.3	1.6	1.289	0.961	4.040 [d]	2.503	2.711
		59.5	7.3		1.4	60.1	1.472	0.88	4.155 [d]	3.198	2.397
		60.2	8		1.8	90.2	1.595	0.895	4.541 [d]	3.519	2.186
	condensed organic matter[45]						0.810	0.716	4.088 [g]	3.527	3.534
							0.940	0.751	4.163 [g]	3.543	3.311
							0.940	0.716	3.998 [g]	3.437	3.311
							0.910	0.723	3.509 [g]	2.937	3.362
							0.920	0.708	4.164 [g]	3.616	3.345
							0.940	0.718	4.294 [g]	3.730	3.311
							0.580	0.652	4.056 [g]	3.586	3.929
							0.850	0.707	4.221 [g]	3.674	3.465
							0.560	0.704	4.042 [g]	4.522	3.963

[a]: ash corrected. The values of log K_f are all ash corrected. The equation that is used for ash correcting is written as log K_f = log K_f' – log (1-η), where K_f' was the published data after unit transformation and η was the ash content (wt.%) in biochars. K_f, K_f': (mg kg^{-1}) (L $mg^{-1})^N$

[b]: log Q_A (exp) represented the experimental logarithmic maximum adsorption capacity generated from the high-concentration linear fitting of two points in the Freundlich equation at 3/4 C_s and C_s equilibrium concentration. C_s refers to the solubility of naphthalene and phenanthrene in water which were 32 mg L^{-1} and 1.15 mg L^{-1} according to reference[10,24], repectively.

[c]: log Q_A (pre) represented the logarithmic maximum adsorption capacity predicted predicted from the fitted linear relationship between H/C and log Q_A.

[d]: Unit transformation: The log K_f' was obtained from the equation (log K_f' = log (K_{foc}*$C\%$)), where K_{foc} was the organic carbon-normalized sorption capacity coefficient with unit of (μg g^{-1} OC) (L $mg^{-1})^N$ in the published article.

[e]: Unit transformation: The log K_f' was obtained from the equation (log K_f' = log K_{foc} - 3 + log ($C\%$) - N*log (5.97)) , where K_{foc} was the organic carbon-normalized

sorption capacity coefficient with unit of (μg kg^{-1} OC) in the published article.

[f]: Unit transformation: The log K_f' was obtained from the equation (log K_f' = log K_{foc} + log ($C\%$) + 3*N), where K_{foc} was the organic carbon-normalized sorption capacity coefficient with unit of (μg g^{-1} OC) (L μg^{-1})^N in the published article.

[g]: Unit transformation: The log K_f' was obtained from the equation (log K_f' = log K_f + 3*N), where K_f was the sorption capacity coefficient with unit of (μg g^{-1}) (L μg^{-1})^N in the published article..

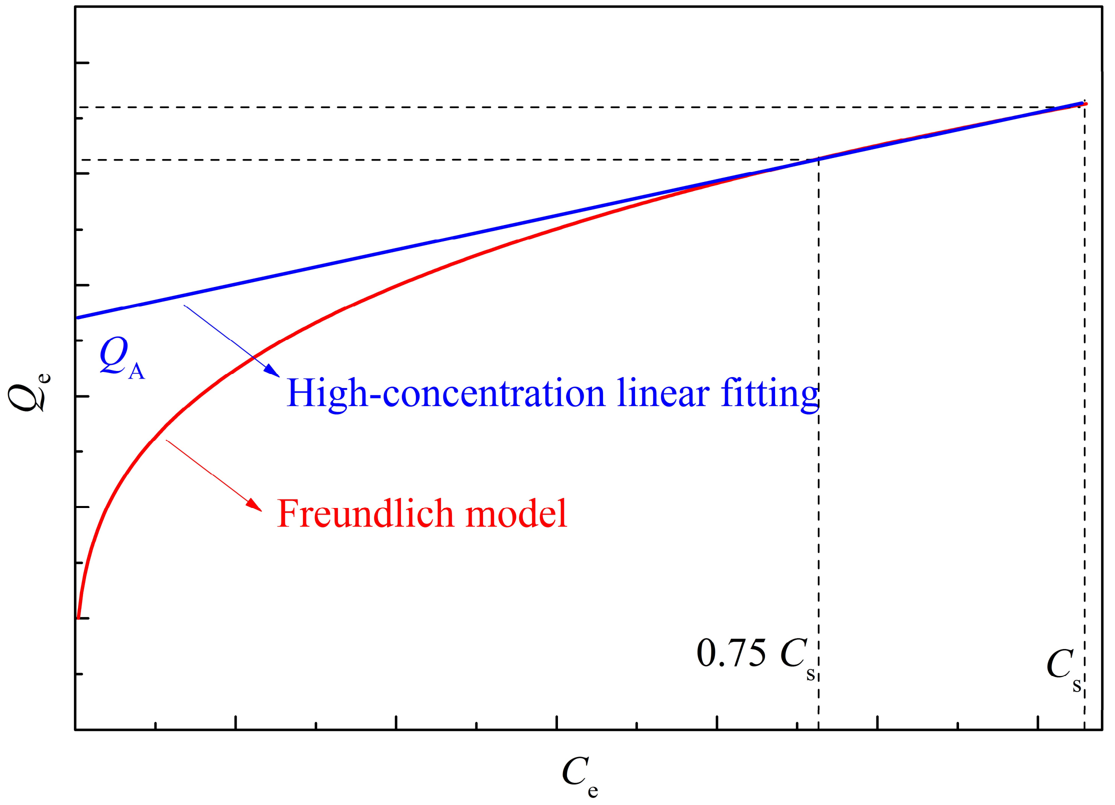

Figure S-1 Illustration of the approximate calculation process of the maximum adsorption capacity from Freundlich parameters using the high-concentration linear fitting method. Two points were used for the high-concentration linear fitting from the Freundlich isotherm at 0.75C_s and C_s. Q_A represented the maximum adsorption capacity. $Q_A = K_f*C_s^N-4*(K_f*C_s^N - K_f*(0.75 C_s)^N)$, C_s represented the sorbate solubility in water.

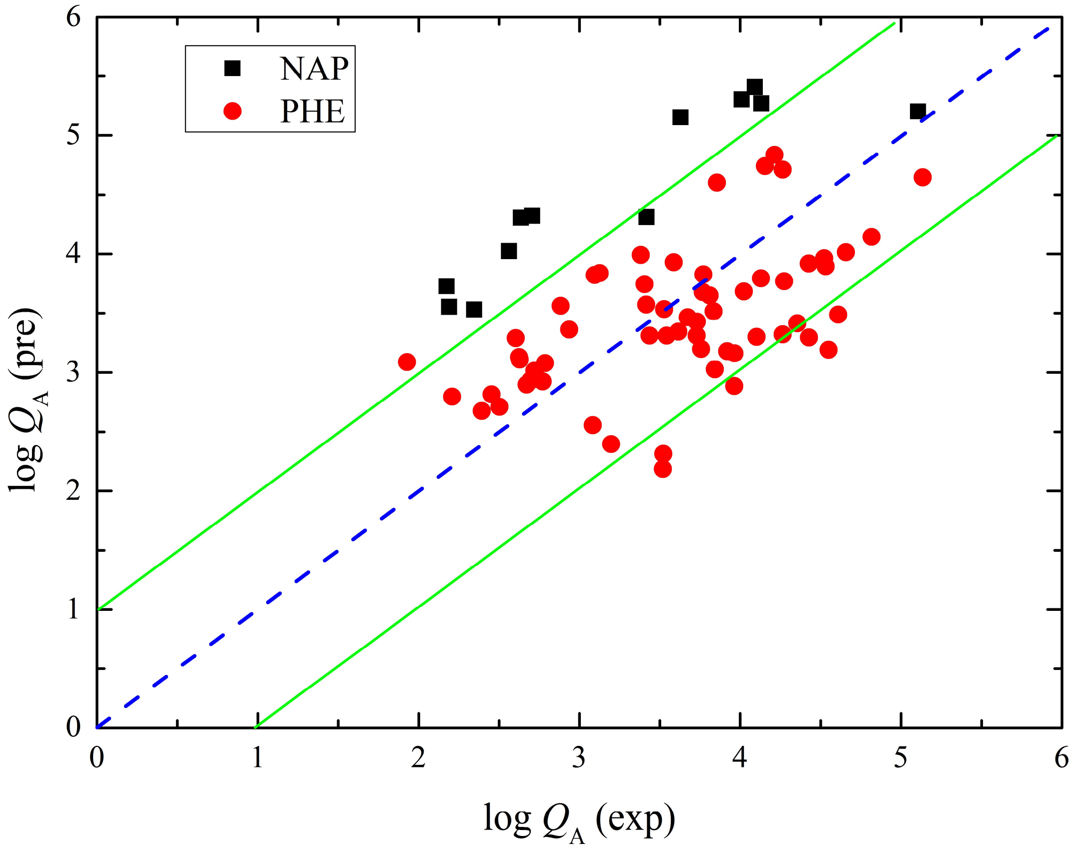

Figure S-2 Relationships of log Q_A (exp) and log Q_A (pre) of organic pollutions onto other carbon materials (not biochars). logQ_A (exp) represented the experimental logarithmic maximum adsorption capacity generated from the high-concentration linear fitting. log Q_A (pre) represented the logarithmic maximum adsorption capacity predicted from the fitted linear relationship between *H/C* and log Q_A. The units of Q_A are mg kg^{-1}. Please refer to Table S-5 for specific data.

References:

1. Liu, P. *et al.* Modification of bio-char derived from fast pyrolysis of biomass and its application in removal of tetracycline from aqueous solution. *Bioresource Technol.* **121**, 235-240 (2012).

2. Liu, Z., Zhang, F. & Wu, J. Characterization and application of chars produced from pinewood pyrolysis and hydrothermal treatment. *Fuel* **89**, 510-514 (2010).

3. Kloss, S. *et al.* Characterization of slow pyrolysis biochars: Effects of feedstocks and pyrolysis temperature on biochar properties. *J. Environ. Qual.* **41**, 990-1000 (2012).

4. Cantrell, K. B., Hunt, P. G., Uchimiya, M., Novak, J. M. & Ro, K. S. Impact of pyrolysis temperature and manure source on physicochemical characteristics of biochar. *Bioresource Technol.* **107**, 419-428 (2012).

5. Uchimiya, M., Wartelle, L. H., Klasson, K. T., Fortier, C. A. & Lima, I. M. Influence of pyrolysis temperature on biochar property and function as a heavy metal sorbent in soil. *J. Agr. Food Chem.* **59**, 2501-2510 (2011).

6. Uchimiya, M., Wartelle, L. H., Lima, I. M. & Klasson, K. T. Sorption of deisopropylatrazine on broiler litter biochars. *J. Agr. Food Chem.* **58**, 12350-12356 (2010).

7. Lin, Y., Munroe, P., Joseph, S., Henderson, R. & Ziolkowski, A. Water extractable organic carbon in untreated and chemical treated biochars. *Chemosphere* **87**, 151-157 (2012).

8. Ro, K. S., Cantrell, K. B. & Hunt, P. G. High-temperature pyrolysis of blended animal manures for producing renewable energy and value-added biochar. *Ind. Eng. Chem. Res.* **49**, 10125-10131 (2010).

9. Lee, J. W. *et al.* Characterization of biochars produced from cornstovers for soil amendment. *Environ. Sci. Technol.* **44**, 7970-7974 (2010).

10. Chen, B., Zhou, D. & Zhu, L. Transitional adsorption and partition of nonpolar and polar aromatic contaminants by biochars of pine needles with different pyrolytic temperatures. *Environ. Sci. Technol.* **42**, 5137–5143 (2008).

11. Keiluweit, M., Nico, P. S., Johnson, M. G. & Kleber, M. Dynamic molecular structure of plant biomass-derived black carbon (biochar). *Environ. Sci. Technol.* **44**, 1247-1253 (2010).

12. Chen, Z., Chen, B. & Zhou, D. Composition and sorption properties of rice-straw derived biochars. *Acta Scientiae Circumstantiae* **33**, 9-19 (2013).

13. Ahmad, M. *et al.* Biochar as a sorbent for contaminant management in soil and water: A review.

Chemosphere **99**, 19-33 (2014).

14. Chen, B. & Chen, Z. Sorption of naphthalene and 1-naphthol by biochars of orange peels with different pyrolytic temperatures. *Chemosphere* **76**, 127-133 (2009).
15. Karaosmanoglu, F., Isigigur-Ergundenler, A. & Sever, A. Biochar from the straw-stalk of rapeseed plant. *Energ. Fuel.* **14**, 336-339 (2000).
16. Lian, F., Huang, F., Chen, W., Xing, B. & Zhu, L. Sorption of apolar and polar organic contaminants by waste tire rubber and its chars in single- and bi-solute systems. *Environ. Pollut.* **159**, 850-857 (2011).
17. Chen, Z., Chen, B. & Chiou, C. T. Fast and slow rates of naphthalene sorption to biochars produced at different temperatures. *Environ. Sci. Technol.* **46**, 11104–11111 (2012).
18. Ahmad, M. *et al.* Effects of pyrolysis temperature on soybean stover- and peanut shell-derived biochar properties and TCE adsorption in water. *Bioresource Technol.* **118**, 536-544 (2012).
19. Mohan, D., Rajput, S., Singh, V. K., Steele, P. H. & Pittman, C. U. Modeling and evaluation of chromium remediation from water using low cost bio-char, a green adsorbent. *J. Hazard. Mater.* **188**, 319-333 (2011).
20. Fang, Q., Chen, B., Lin, Y. & Guan, Y. Aromatic and hydrophobic surfaces of wood-derived biochar enhance perchlorate adsorption via hydrogen bonding to oxygen-containing organic groups. *Environ. Sci. Technol.* **48**, 279-288 (2014).
21. Wang, X. & Xing, B. Sorption of organic contaminants by biopolymer-derived chars. *Environ. Sci. Technol.* **41**, 8342-8348 (2007).
22. Xu, Y. & Chen, B. Organic carbon and inorganic silicon speciation in rice-bran-derived biochars affect its capacity to adsorb cadmium in solution. *J. Soil. Sediment.*, 1-11 (2014).
23. Mullen, C. A. *et al.* Bio-oil and bio-char production from corn cobs and stover by fast pyrolysis. *Biomass Bioenerg.* **34**, 67-74 (2010).
24. Zhang, M. *et al.* Characterization of nitrogen-rich biomaterial-derived biochars and their sorption for aromatic compounds. *Environ. Pollut.* **195**, 84-90 (2014).
25. Lattao, C., Cao, X., Mao, J., Schmidt-Rohr, K. & Pignatello, J. J. Influence of molecular structure and adsorbent properties on sorption of organic compounds to a temperature series of wood chars. *Environ. Sci. Technol.* **48**, 4790-4798 (2014).
26. Huang, H., Wang, Y.-x., Tang, J.-c. & Zhu, W.-y. Properties of maize stalk biochar produced under

different pyrolysis temperatures and its sorption capability to naphthalene. *Environmental Science* **35**, 1884–1890 (2014).

27. Chen, B. & Yuan, M. Enhanced sorption of polycyclic aromatic hydrocarbons by soil amended with biochar. *J. Soil. Sediment.* **11**, 62-71 (2011).

28. Sun, K. *et al.* Impact of deashing treatment on biochar structural properties and potential sorption mechanisms of phenanthrene. *Environ. Sci. Technol.* **47**, 11473-11481 (2013).

29. Valili, S., Siavalas, G., Karapanagioti, H. K., Manariotis, I. D. & Christanis, K. Phenanthrene removal from aqueous solutions using well-characterized, raw, chemically treated, and charred malt spent rootlets, a food industry by-product. *J. Environ. Manage.* **128**, 252-258 (2013).

30. Qiu, M. *et al.* Properties of the plant- and manure-derived biochars and their sorption of dibutyl phthalate and phenanthrene. *Sci. Rep.* **4**, 5295 (2014).

31. Han, L. *et al.* Role of structure and microporosity in phenanthrene sorption by natural and engineered organic matter. *Environ. Sci. Technol.* **48**, 11227-11234 (2014).

32. Jin, J. *et al.* Single-solute and bi-solute sorption of phenanthrene and dibutyl phthalate by plant- and manure-derived biochars. *Sci. Total Environ.* **473**, 308-316 (2014).

33. Wu, M. *et al.* The sorption of organic contaminants on biochars derived from sediments with high organic carbon content. *Chemosphere* **90**, 782-788 (2013).

34. Chen, N. *et al.* Sorption and desorption of phenanthrene in the biochar derived from Dianchi sediment. *Environ. Chem.* **30**, 2026-2031 (2011).

35. Sun, K. *et al.* Sorption of bisphenol A, 17 alpha-ethinyl estradiol and phenanthrene on thermally and hydrothermally produced biochars. *Bioresource Technol.* **102**, 5757-5763 (2011).

36. Xing, B. Sorption of naphthalene and phenanthrene by soil humic acids. *Environ. Pollut.* **111**, 303-309 (2001).

37. Wang, X., Lu, J. & Xing, B. Sorption of organic contaminants by carbon nanotubes: Influence of adsorbed organic matter. *Environ. Sci. Technol.* **42**, 3207-3212 (2008).

38. Wang, J., Chen, Z. & Chen, B. Adsorption of polycyclic aromatic hydrocarbons by graphene and graphene oxide nanosheets. *Environ. Sci. Technol.* **48**, 4817-4825 (2014).

39. Zhang, J. & He, M. Effect of structural variations on sorption and desorption of phenanthrene by sediment organic matter. *J. Hazard. Mater.* **184**, 432-438 (2010).

40. Pan, B. *et al.* Effect of physical forms of soil organic matter on phenanthrene sorption.

Chemosphere **68**, 1262-1269 (2007).

41. Yang, C. *et al.* Intercorrelations among degree of geochemical alterations, physicochemical properties, and organic sorption equilibria of kerogen. *Environ. Sci. Technol.* **38**, 4396-4408 (2004).
42. Yan, C., Yang, Y., Liu, M., Nie, M. & Zhou, J. L. Phenanthrene sorption to Chinese coal: Importance of coal's geochemical properties. *J. Hazard. Mater.* **192**, 86-92 (2011).
43. Zhang, J., He, M., Lin, C. & Shi, Y. Phenanthrene sorption to humic acids, humin, and black carbon in sediments from typical water systems in China. *Environ. Monit. Assess.* **166**, 445-459 (2010).
44. Kang, S. & Xing, B. Phenanthrene sorption to sequentially extracted soil humic acids and humins. *Environ. Sci. Technol.* **39**, 134-140 (2005).
45. Ran, Y., Sun, K., Yang, Y., Xing, B. & Zeng, E. Strong sorption of phenanthrene by condensed organic matter in soils and sediments. *Environ. Sci. Technol.* **41**, 3952-3958 (2007).
